# Greenhouse Gas Reductions Driven by Vehicle Electrification across Powertrains, Classes, Locations, and Use Patterns

**DOI:** 10.1021/acs.est.5c05406

**Published:** 2025-08-25

**Authors:** Elizabeth Smith, Maxwell Woody, Timothy J. Wallington, Christian Hitt, Hyung Chul Kim, Alan I. Taub, Gregory A. Keoleian

**Affiliations:** † Electric Vehicle Center, 1259 University of Michigan, Ann Arbor, Michigan 48109, United States; ‡ Center for Sustainable Systems, School for Environment and Sustainability, University of Michigan, 440 Church Street, Ann Arbor, Michigan 48019, United States; § Mechanical Engineering, University of Michigan, 2350 Hayward St, Ann Arbor, Michigan 48124, United States; ∥ Materials Science and Engineering, University of Michigan, 515 E. Jefferson St, Ann Arbor, Michigan 48109, United States; ⊥ Research and Innovation Center, 1931 Ford Motor Company, Dearborn, Michigan 48121, United States

**Keywords:** life cycle assessment, light duty vehicles, transportation decarbonization, battery electric vehicles, plug-in hybrid electric vehicles, hybrid electric vehicles, GHG emissions calculator and maps

## Abstract

We assess the cradle-to-grave greenhouse gas (GHG) emissions of current (2025) light-duty vehicles (LDV) across powertrains, vehicle classes, and locations. We create driver archetypes (commuters, occasional long-distance travelers, contractors), simulate different use patterns (drive cycles, utility factors, cargo loads) and characterize GHG emissions using an attributional approach. Driven by grid decarbonization and improved electric vehicle efficiency, we are first to report electric vehicles have lower GHG emissions than gasoline vehicles in every county across the contiguous United States. On average, a 300-mile range battery electric vehicle (BEV) has emissions which are 31–36% lower than a 50-mile range plug-in hybrid electric vehicle (PHEV), 63–65% lower than a hybrid electric vehicle (HEV), and 71–73% lower than an internal combustion engine vehicle (ICEV). Downsizing also reduces emissions, with a compact ICEV having 34% lower emissions than an ICEV pickup. We present the first evaluation of LDV emissions while hauling cargo, showing that carrying 2500 lbs. in a pickup increases BEV emissions by 13% (134 to 152 g CO_2_e/mile) compared to 22% (486 to 592 g CO_2_e/mile) for an ICEV. Emissions maps and vehicle powertrain/class matrices highlight the interplay between vehicle classes, powertrains, locations, and use patterns, and provide insights for consumers, manufacturers, and policymakers.

## Graphical abstract

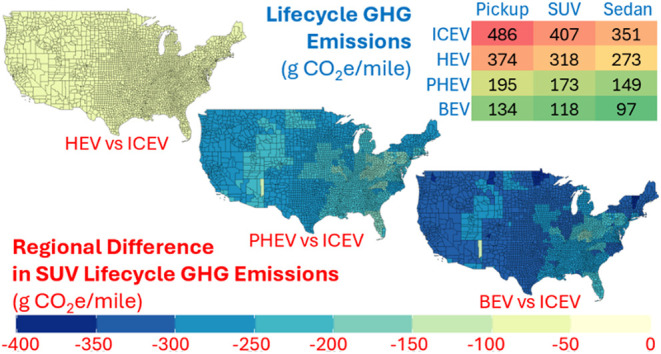

## 1 Introduction

Electrification of the transportation sector is crucial to meet national climate targets as the sector makes up 28% of United States greenhouse gas (GHG) emissions, with 57% of those emissions coming from light duty vehicles (LDVs).^1^ Consumer transportation choices, including vehicle selection and usage patterns, will play a significant role in reducing GHG emissions in the transportation sector.

Vehicle class and powertrain are critical parameters that shape GHG emissions. Electrification of powertrains is a powerful tool for decarbonization. Battery electric vehicles (BEVs) have substantial environmental benefits over both internal combustion engine vehicle (ICEV) and hybrid electric vehicle (HEV) alternatives, but have disadvantages such as limited range, inadequate charging infrastructure, longer refueling times, and performance issues in extreme environments.^2,3^ BEVs have a larger production burden than other powertrains due to the need for critical minerals and substantial energy for manufacturing long-range batteries.^4^ Plug-in hybrid electric vehicles (PHEVs) combine electric and gasoline (or diesel) powertrains removing range anxiety concerns and reducing the need for extensive mining required for larger battery packs. PHEVs offer many of the benefits of BEVs with the added flexibility of being able to use conventional fuel. To inform consumers, the automotive industry, and national decarbonization strategies, it is important to have a detailed understanding of the lifecycle emissions associated with different vehicle classes, powertrains, and use patterns across the U.S.

Previous research has explored various aspects of vehicle class and powertrain comparisons, particularly focusing on average emissions associated with cradle-to-gate and use phases of ICEVs and BEVs. Studies of PHEVs across a range of low and high carbon grids have been reported, with most research centered on sedans.^5–12^ Relatively few studies analyze SUVs or pickup trucks which are the most popular vehicle classes in the U.S.^13,14^

When estimating lifecycle emissions of PHEVs and BEVs across the U.S., it is important to account for different electricity grid emissions and ambient temperatures. Few studies use county-level estimates for electricity grid calculations and temperature effects,^14,15^ with many relying on broader NERC regions instead and standard on-road fuel economy. Several studies have accounted for temperature differences between counties using different techniques, though only one included PHEVs in the analysis.^15^ Often studies have employed EPA labels or CAFE standards to reflect fuel economy adjustments.^5,6,12,15–18^

Emissions from the U.S. electricity grid have decreased substantially over the past 5–10 years and are expected to continue to decline reflecting progress in renewable energy development and deployment.^19^ Most literature life cycle assessment (LCA) studies of EVs in the U.S. are based on historical grid emission factors which may underestimate the benefits of electrification.

When assessing lifecycle emissions, it is important to consider different vehicle use cases. Previous studies have examined the effect of the utility factor of PHEVs, most using standards set by vehicle all-electric-range,^5,17^ and some using national travel data to derive utility factors by state.^14,20^ One study compared three driving scenarios: all-electric, all-gasoline, and a mixed scenario with 80% electric driving.^16^ While many studies have used standard fuel economies from the EPA or manufacturer information,^12,16,17^ none have examined the implications of drive cycle on different user profiles or in conjunction with added cargo. Hauling cargo is an important use for pickup trucks. While significant research has focused on the benefits of lightweighting vehicles,^17,21–23^ there have been no studies of the impact of cargo on GHG emissions for light-duty vehicles.

Both attributional and consequential life cycle assessment have been used to compare the greenhouse gas emissions of different vehicles.^24^ Attributional LCA aims to assign or apportion emissions to products or systems. Consequential LCA aims to estimate the emissions resulting from a change to a system or policy, or a decision between products. Each method answers different, though related, research questions. For example, attributional LCA could be used to assign emissions to different existing powertrains as they contribute to overall transportation sector emissions. Consequential LCA could be used to estimate the change in net emissions resulting from a switch from one powertrain to another.

Most literature studies are attributional, some are consequential, and very few studies use both approaches. Those that do (e.g., Tamayao et al., 2015, Woody et al., 2023, Singh et al., 2024) find that consequential assessments have significantly higher GHG emission estimates for electric vehicles.

To address the shortcomings described above, we present a comprehensive lifecycle parametric model to assess GHG emissions of vehicles across different powertrains (ICEV, HEV, PHEV, BEV), classes (sedan, SUV, pickup truck) and use patterns (utility factor, drive cycle, and cargo). We analyze geographical heterogeneity at the county level accounting for local temperature and driving pattern effects. We use both attributional and consequential approaches for future grid burdens. Driven mainly by grid decarbonization but also by improved electric vehicle efficiency, we are the first to find that vehicle electrification reduces GHG emissions in every county across the contiguous United States using both attributional and consequential LCA methods. Our results showcase the relative environmental performance of different vehicles under various scenarios to help inform consumers, policymakers, and manufacturers in decarbonizing light duty transportation.

## 2 Methods

### 2.1 Scope

This study compares driver archetypes to estimate the GHG emissions of different vehicles and use patterns. The framework builds upon a lifecycle assessment model that evaluates the cradle to grave GHG impact associated with different vehicle powertrains (ICEVs, HEVs, PHEVs, and BEVs), vehicle classes (compact and midsize sedans, small and midsize SUVs, and pickup trucks), and use patterns (utility factors, drive cycles, and cargo loads). The vehicle models used in this study are generic and constructed to inform consumers, policy makers, and the automotive industry about vehicle lifecycle GHG emissions.

For the baseline analysis we employ an attributional approach using average emissions rates (AER) from NREL's Cambium 2023 Midcase scenario.^32^ The Cambium model assumes grid electricity demand growing at approximately 1.8% per year and accounts for projected EV adoption (from 20 TWh in 2024 to 250 TWh in 2030), and we assume no transmission and distribution expansion beyond what is accounted for Cambium. We compare the attributional life cycle emissions for the different ICEV, HEV, PHEV, and BEV options.^25^ While our baseline analysis employs an attributional approach, we also consider a consequential approach and present results using short- and long-run marginal emission rates for electricity generation from the Cambium model in Section 3.3.

### 2.2 Vehicle Cycle

To ensure a consistent comparison of generic vehicles, we model outputs such as fuel economy, battery size, and curb weight from the Argonne National Laboratory Autonomie model, which simulates vehicle energy consumption across vehicle classes, powertrains, and levels of future technology development.^26^ We include BEVs with 200-, 300- and 400-mile ranges, PHEVs with 35- and 50-mile ranges, and gasoline ICEVs and HEVs across five vehicle classes (SI Table S4). These parameters are compared to actual vehicles in SI Figure 2.

Vehicle cycle emissions (materials, manufacturing, and end-of-life) are calculated using the GREET 2023 model from Argonne National Laboratory.^27^ We modified battery size and curb weight for each of the corresponding Car, SUV and Pickup options using vehicle parameters for model year 2025 (SI Note 2). Vehicle cycle emissions include production of components and fluids over the vehicle lifetime along with assembly and disposal of the vehicle. We do not include Li-ion battery replacements during the vehicle lifetime. The latest data shows that for new models, batteries tend to outlast the vehicle’s useful life.^28^ We assumed a battery chemistry of NMC811 for BEV, PHEV, and HEV as an example of a high-nickel chemistry, the most common chemistry in the current U.S. EV market.^29^ For completeness, the impact of assuming NMC111, NMC622, or LFP battery chemistry is also explored and discussed in SI Note 7. The total emissions for vehicles with these other battery chemistries differ by less than 2.5% from those with NMC811.

### 2.3 Use Phase

We assume electrified and nonelectrified vehicles have the same lifetime vehicle miles traveled (VMT): 191,386 miles for sedans, 211,197 miles for SUVs, and 244,179 miles for pickup trucks in our baseline scenario.^30^ The lifecycle use phase emissions for ICEV and HEV are calculated using eq 1.

1 $$\mathrm{GH}G_{\mathrm{ICEV},\mathrm{HEV}}=\sum_{y=2025}^{y+L_{v}}\frac{M_{y}\times \mathrm{CI}}{FE_{g}}$$

Annual emissions are calculated starting at year *y* and ending *L*_*v*_ years later, where *L*_*v*_ is the lifetime of the vehicle. The annual miles driven, *M*_*y*_, in year *y* follows data by the National Highway Traffic and Safety Association (SI Figure S3).^30^ Fuel economy of the gasoline vehicles (FE_*g*_) is measured in miles/gallon. The carbon intensity, CI, is the well-to-wheel (WTW) carbon intensity of gasoline which includes all upstream impacts (e.g., refining) (10.647 kg CO_2_e/gallon).^31^

Lifecycle use phase emissions for the BEV are calculated using eq 2.

2 $$\mathrm{GH}G_{\mathrm{BEV}}=\sum_{y=2025}^{y+L_{v}}\frac{M_{y}\times {\mathrm{FC}}_{e}\times {\mathrm{EF}}_{y}}{\eta }$$

Fuel consumption of the electric powered vehicle, FC, is measured in $\left[\frac{\mathrm{Wh}}{\mathrm{mile}}\right]$ . An emissions factor, EF_*y*_, of the electricity grid in year *y* is applied for every year over the life of the vehicle $\left[\frac{\mathrm{gC}O_{2}e}{\mathrm{kWh}}\right]$ . We used NREL’s Cambium 2023 Midcase scenario, which predicts future changes in grid carbon intensity based on current policies.^32^ We used annual average value for emissions factors from 2025 through the life of the vehicle and a charging efficiency, η, of 88%.^31^ Fuel economy (FE_g_) and fuel consumption (FC_e_) values are sourced from Autonomie.

To calculate use phase emissions for PHEVs, we use an average of the two equations above weighted by the fraction of miles in electric mode (utility factor, UF). We assume that when in charge- sustaining and charge-depleting mode, PHEVs function similarly to HEVs and BEVs, respectively.

3 $$\mathrm{GH}G_{\mathrm{PHEV}}=\left(\mathrm{UF}\times \mathrm{GH}G_{\mathrm{BEV}}\right)+\left(\left(1-\mathrm{UF}\right)\times \mathrm{GH}G_{\mathrm{HEV}}\right)$$

GHG_BEV_ and GHG_HEV_ are the annualized values for GHG emissions over the lifetime of the vehicle. Baseline utility factors for the PHEV35 and PHEV50 are 58 and 69%, respectively, based on the SAE Standard.^33^ Unless otherwise specified, use phase calculations assume typical driving which is defined by a standard city/highway split of 43/57, SAE standard utility factors, a set VMT schedule, and no cargo (SI Note 2).

### 2.4 Regional Variation

We analyzed the impacts of different vehicles across the U.S. at a county level. Grid carbon intensities vary greatly across the country as some states rely heavily on coal and natural gas to power their grid, while others have adopted, or plan to adopt, high levels of renewable energy. Variability in the grid was accounted for using 134 balancing areas designated in the NREL Cambium model.^32^ A key reason for using balancing areas instead of larger eGRID subregions is the substantial differences in emissions factors. For example, the balancing areas in southwest Minnesota (BA44) and northwestern Iowa (BA45) both fall under the MROW eGRID subregion and are geographically adjacent. However, Minnesota (BA44) has an emissions factor of 41 kg CO_2e_/MWh, while Iowa (BA45) has 382 kg CO_2e_/MWh in 2025. These disparities underscore the importance of using more granular balancing area data. Cambium explicitly accounts for imports and exports between balancing areas, incorporating interregional trade to ensure accurate load calculations, as noted in its documentation.^32^ Also studies have shown that greater spatial resolution can better reflect regional boundaries in the grid.^34,35^ Ambient temperature has a larger impact on range and efficiency of electric vehicles than for conventional ICEVs. At temperatures below 20 °F, BEV can lose up to 40% of their range and fuel economy compared to 75 °F ambient temperature.^3^ We obtained the average monthly temperature for each county over the past 5 years (2019–2023) from NOAA.^36^ The effect of temperature on fuel economy was calculated following the work of Wu et al. (SI Note 5).^37^

### 2.5 Cargo and Fuel Reduction Values

Carrying cargo is an important vehicle function that has not been included in any previously reported light duty vehicle LCA. We explore the effect of cargo on GHG emissions for pickup trucks, building on previous work and providing new analysis of existing data from the physics-based model of Kim et al. to calculate fuel reduction values (FRVs) for different powertrains. This model was developed to quantify the fuel consumption changes from vehicle lightweighting.^22^ We present its first use to estimate the impact of cargo on emissions.

FRV gives the increase in fuel consumption when driving 100 km with 100 kg of weight added to the vehicle $\left[\frac{L_{\mathrm{gas}‐\mathrm{eq}}}{100\;\mathrm{km}\times 100\;\mathrm{kg}}\right]$ . Table 1 shows the FRVs derived from Kim et al. corresponding to each Autonomie vehicle used in the present study (SI Figure S5). FRVs are dependent on several factors including drive cycle, vehicle size and power, powertrain configuration and efficiency. Our estimated FRVs are largely consistent with literature values.^38,39^ For example, Del Pero et al. determined FRV of 0.055–0.078L/(100 km 100 kg) for BEVs under the WLTP cycle.^39^ For ICEVs, Geyer & Malen determined FRV of 0.16–0.17 L/(100 km 100 kg) under the US combined driving cycle.^38^ This agrees with our result considering that on-road adjusted fuel economy is ∼70% of unadjusted fuel economy.^40^

**Table 1:** Fuel Reduction Values (FRVs) for Autonomie Pickup Trucks with Different Powertrains Derived from Kim et al. CS = Charge Sustaining Mode, CD = Charge Depleting Mode

powertrain	FRV highway (liter eq/100 km × 100 kg)	FRV city (liter eq/100 km × 100 kg)	FRV combined (liter eq/100 km × 100 kg)
ICEV	0.247	0.189	0.216
HEV	0.125	0.140	0.133
PHEV35-CS	0.120	0.139	0.130
PHEV50-CS	0.120	0.139	0.130
PHEV 35-CD	0.085	0.075	0.080
PHEV50-CD	0.085	0.077	0.081
BEV200	0.062	0.053	0.057
BEV300	0.062	0.053	0.057
BEV400	0.064	0.053	0.058

The FRV for vehicle powertrain *p* and drive cycle *d* (urban or highway) was used to calculate the additional fuel consumption for cargo weight (*W*), measured in [kg].

4 $${\mathrm{FC}}_{W}={\mathrm{FRV}}_{p,d}\times W/100$$

The additional fuel consumption (FC_*W*_), measured in $\left[\frac{L_{\mathrm{gas}‐\mathrm{eq}}}{100\;\mathrm{km}}\right]$ was added to the unloaded vehicle fuel consumption (FC_gas/elec_) $\left[\frac{L_{\mathrm{gas}‐\mathrm{eq}}}{100\;\mathrm{km}}\right]$ to get the fuel consumption with cargo (FC_Loaded_) which was used in the model.

5 $${\mathrm{FC}}_{\mathrm{loaded}}={\mathrm{FC}}_{\mathrm{gas}/\mathrm{elec}}+{\mathrm{FC}}_{W}$$

Table 1 presents the regression analysis results for fuel reduction values (FRV) across different powertrain types and drive cycles (highway/city) based on data in the Kim et al. study.^22^ Gasoline powertrains are more affected by changes in weight compared to electric powertrains. Similarly, the fuel economy for city driving is more impacted by changes in weight compared to highway driving.

## 3 Results and Discussion

We first examine lifecycle emissions of different powertrains and vehicle classes on a national level. We examine the effects of drive cycle, utility factor, and cargo. We develop vehicle powertrain/class matrices to compare lifecycle GHG emissions for different vehicles.

### 3.1 National Case Lifecycle Emissions

To calculate lifecycle emissions, we start with the production and end-of-life burdens, which are defined as vehicle cycle emissions. As shown in SI Figure S6, vehicle cycle emissions increase as the vehicle is more electrified. This effect is largely due to the impact of batteries. The battery accounts for 48–56% of vehicle cycle emissions for the BEV300 compared to 1% for the ICEV.

Use-phase emissions are 92 and 89% of the lifecycle emissions for ICEVs and HEVs, respectively. In contrast, use-phase for BEVs and PHEVs make up about 48–60 and 73–80% of lifecycle emissions, depending on battery size (SI Figure S6). For use phase calculations, we assume typical driving which is defined by a standard city/highway split of 43/57, SAE standard utility factors for PHEV defined by battery size (58% for PHEV35, 69% for PHEV50), a set VMT schedule, and no cargo.

Figure 1 shows national case emissions relative to other classes and powertrains. We define our national case as a driver with a typical driving profile (standard city/highway split of 43/57, SAE standard utility factors, a set VMT schedule, and no cargo) and national average grid emissions. Vehicle powertrain and class combinations can lead to emissions differences of up to 83% for the extreme example of comparing an ICEV Pickup with a BEV200 Compact Sedan (Figure 1a). Emissions decrease with smaller vehicle classes and with more electrified powertrains. Our analysis shows that powertrain electrification provides greater potential for lifecycle emissions reductions compared to downsizing alone. Figure 1b shows the impact of electrification within each vehicle class, with BEVs and PHEVs having lifecycle emissions up to 68% lower than HEVs and up to 75% lower than ICEVs. Emissions increase with battery size for BEVs, but the opposite is seen in PHEVs since UF increases with battery size. Figure 1c shows the benefit of vehicle downsizing with reductions up to 34% from a pickup to a compact sedan. The emissions benefits from downsizing are less than those from electrification. Electrification has a similar fractional impact across classes, and downsizing has a similar fractional impact across powertrains. Pickup trucks have the largest absolute benefit from electrification and ICEVs have the largest absolute benefit from downsizing due to their higher baseline emissions.

**Figure 1:**
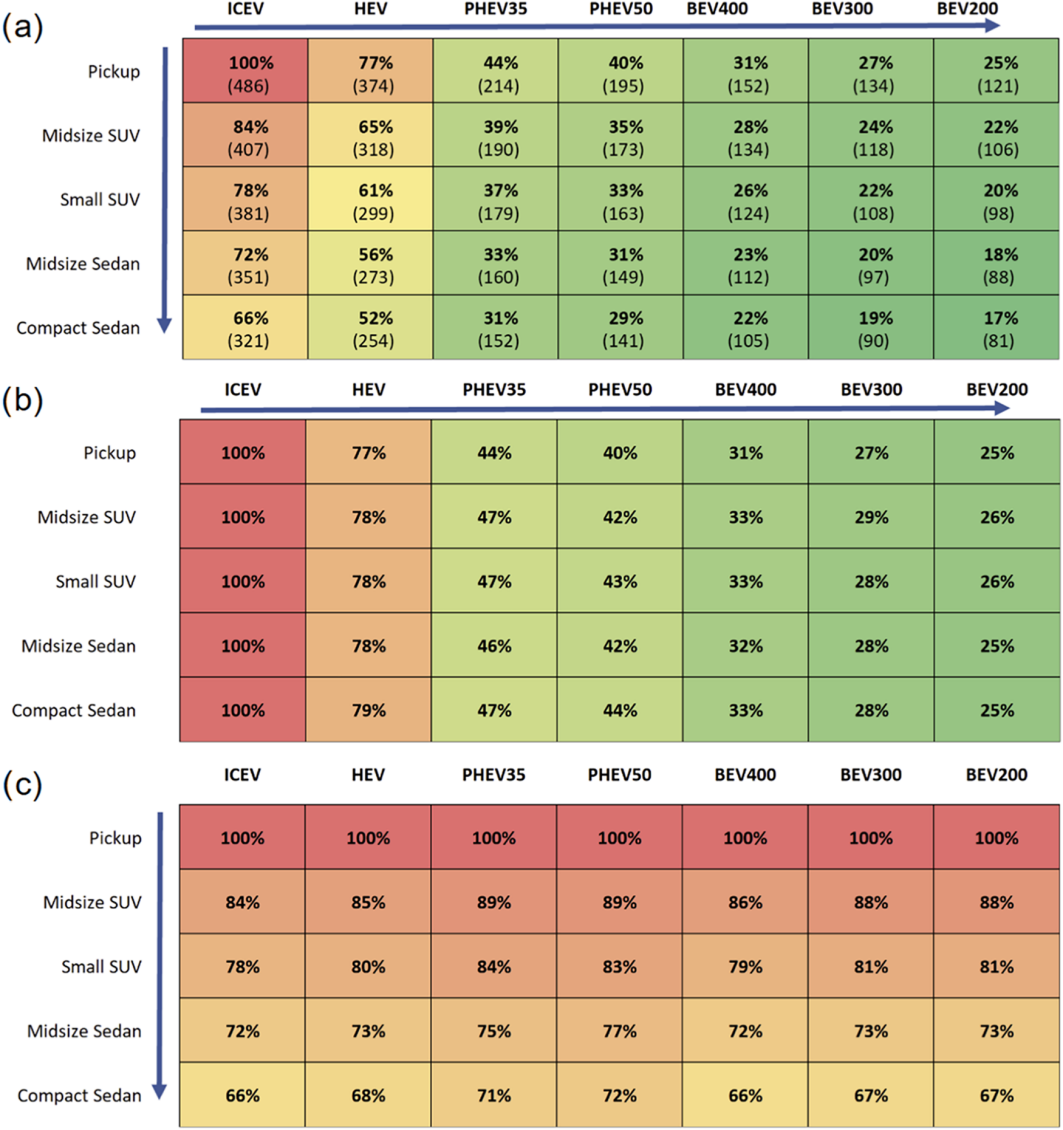
National case lifecycle emissions across vehicle classes and powertrains. Values in parentheses are in units of g CO_2_e/mile, % values are relative to (a) the ICEV pickup (b) the ICEVs of each vehicle class (c) the pickups of each powertrain. Arrows depict direction of GHG reductions. Typical driving profile: city/highway split of 43/57, (UF = 58% for PHEV35; 69% for PHEV50), and no cargo.

### 3.2 Use Patterns and Driver Archetypes

We analyze different use patterns including utility factor, drive cycle, and carrying cargo to encapsulate a wide variety of potential use cases. There are three user archetypes we discuss in this section. (1) A commuter who uses the vehicle solely for commuting or errands. Use patterns that most affect the commuter’s environmental impact are the UF and drive cycle. (2) An occasional long-distance traveler who occasionally uses their vehicle for long trips, but whose daily travel can be met by a small battery, denoting a high UF outside of long trips. In this case, the use pattern that most affects the environmental footprint is the distance traveled, which significantly affects the UF of the PHEV. (3) A contractor, or someone who drives a pickup truck with varying amounts of added cargo. For this analysis, we compare Seattle, WA, and Cincinnati, OH, to highlight grid intensity extremes. Cincinnati, heavily reliant on coal, is in the 96th percentile of U.S. grid carbon intensities, while Seattle, primarily powered by hydropower, is in the third percentile.

#### 3.2.1 Utility Factor

Figure 2 illustrates the effect of utility factor in two different cities for a compact sedan. The slope of the plot is steeper in Seattle because more emissions are offset with every additional mile traveled in charge-depleting mode. Due to this, and the difference in production burden emissions between BEVs and PHEVs, there is a breakeven utility factor between PHEVs and BEVs in Seattle of about 90–99%. In grids with higher carbon intensity, PHEVs have lower emissions than the 400-mile range BEV (for UF > 92%). The PHEV50 at 100% UF only has 16g CO_2_e/mile higher emissions than the BEV300.

**Figure 2:**
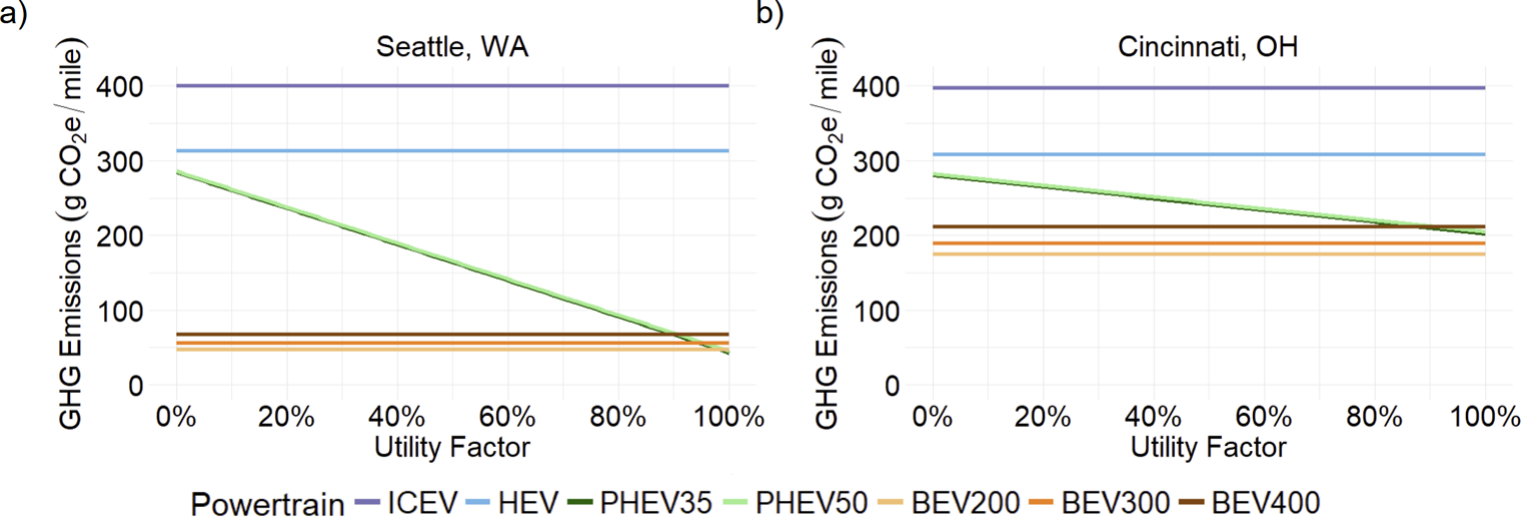
Lifecycle GHG emissions (g CO_2_e/mile) of a compact sedan in (a) Seattle, WA and (b) Cincinnati, OH versus utility factor. Cincinnati and Seattle represent the effects of electricity grid intensities. The utility factor is the percentage of miles driven in electric mode.

UFs for users in areas with cleaner grids have a bigger impact on vehicle lifecycle emissions than for users in dirtier grids. In areas with cleaner grids, BEVs or PHEVs with high UFs offer significant emissions reductions over other powertrain alternatives. For users in dirtier grids, the difference in GHG emissions between BEV, PHEV and HEV is smaller and the influence of the PHEV UF is less pronounced.

In Figure 3, we show results for the vacationer, a special use case of a consumer with a high UF for daily driving but with occasional long distance road trips. These long trips are assumed to use charge sustaining mode on highways. It is assumed that otherwise normal everyday driving needs can be met by charge depleting mode of the PHEV. For the midsize SUV shown in Figure 3, the maximum 10,000 mi/year of long-distance travel would represent ∼75% of total VMT. The more miles traveled long distance in Seattle has a much larger impact on lifecycle emissions than in Cincinnati. BEVs and PHEVs have much lower lifecycle emissions than HEVs and, depending on the amount of long-distance travel per year, BEVs may have significantly lower GHG emissions than PHEVs.

**Figure 3:**
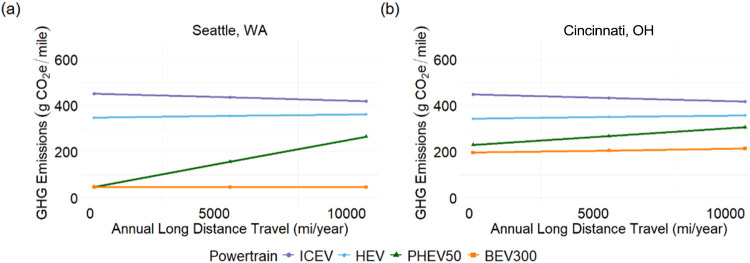
Lifecycle GHG emissions (g CO_2_e/mi) of a midsize SUV versus the annual distance traveled in long trips which are beyond the PHEV all-electric range (a) Seattle, WA and (b) Cincinnati, OH. Long-trip miles are assumed to be driven fully on the highway and, for the PHEV, 100% in gasoline mode. All other miles are assumed to be driven with a 43/57 city/highway split and, for the PHEV, in 100% electric mode.

**Figure 4:**
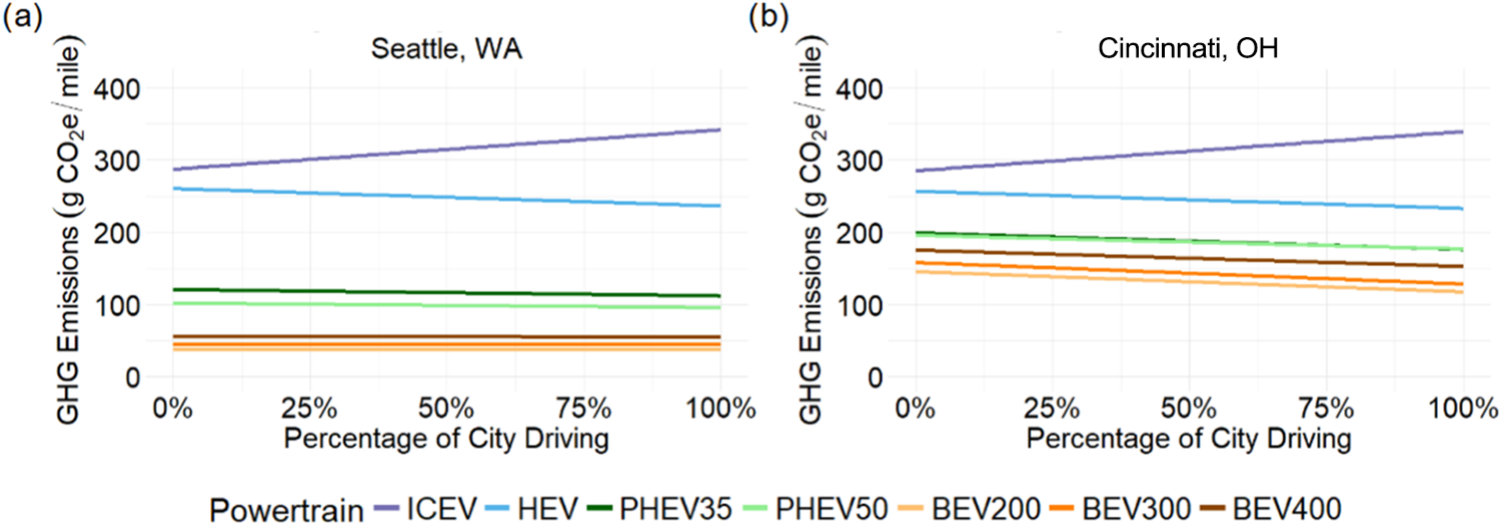
GHG emissions (g CO_2_e/mile) of a compact sedan versus portion of city driving across different powertrains in (a) Seattle, WA and (b) Cincinnati, OH.

#### 3.2.2 Drive Cycle

Drive cycle depends widely on the layout of the city/suburb/rural area and the travel patterns of the driver. Figure 4 shows, for two different cities, as the portion of city driving increases, the benefits of electrified vehicles over ICEVs increase. Drive cycle alone does not change vehicle ranking based on lifecycle GHG emissions. However, in locations with dirtier grids, the effect of increased city driving is more pronounced as BEVs are more efficient at urban than highway driving, and the high grid carbon intensities exacerbate the difference. The difference is also notable for HEVs, and PHEVs compared to ICEVs. This is due to the added benefit of regenerative braking which has a greater impact on city than highway driving. In dirty grids like Cincinnati, OH, PHEVs have somewhat lower emissions than HEVs; in cleaner grids these emissions reductions are much greater.

#### 3.2.3 Cargo

Figure 5 shows the effect of cargo on the national case lifecycle emissions for pickup trucks with different powertrains. The addition of cargo has a greater GHG impact on ICEVs than on the BEV200, about 22 and 13% respectively. This is due to regenerative braking and the relative powertrain efficiencies which reduce the impact of additional mass. Drivers may perform a significant amount of driving unloaded. The unloaded emissions can be used to estimate average footprint based on the typical load and % of unloaded driving (e.g., an ICEV which drives 50% loaded with 2500 lbs and 50% unloaded would have an average emissions rate of 539 g/mile).

**Figure 5:**
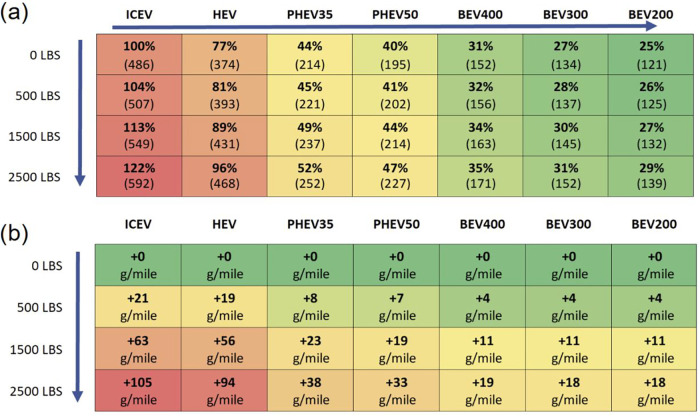
Effects of cargo on lifecycle emissions (g CO_2_e/mile) for pickup trucks with different powertrains. Panel (a) shows results relative to the unloaded ICEV with loaded vehicle g CO_2_e/mile values shown in parentheses. Panel (b) shows results in g CO_2_e/mile relative to the unloaded powertrains. Due to the change in units each heat map is on its own scale. City/highway split of 43/57, UF = 58% for PHEV35; 69% for PHEV50, national grid emissions and temperature.

Figure 6 compares the effect of cargo weight on emissions and is relevant for the contractor use case. Renewable energy on the grid has a significant impact on the increase in emissions for each powertrain from added cargo. In Cincinnati, the emissions from the BEV300 increase by 20% (+43 g CO_2_e/mile) with 2500 lbs of cargo while in Seattle the emissions increase by only 1% (+0.5 g CO_2_e/mile). ICEV emissions increase by 23% (+111 g CO_2_e/mile) for the same load in both locations. The impact of cargo on emissions from HEVs and PHEVs falls between those for ICEVs and BEVs (see Figure 6).

**Figure 6:**
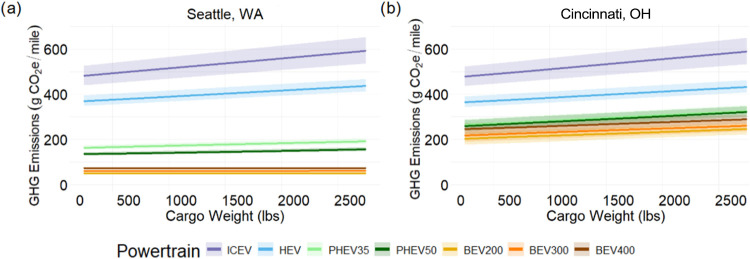
Lifecycle GHG emissions (g CO_2_e/mile) versus cargo for a pickup truck in (a) Seattle, WA, and (b) Cincinnati, OH. Cincinnati and Seattle represent the effects of electricity grid intensities. The shaded region illustrates the effect that drive pattern has on emissions. For ICEV, city driving has much higher emissions than all highway driving. For all other electrified vehicles, highway driving has more impact than city driving, but to a much lesser extent. This is due to regenerative braking utilized by electrified vehicles.

In regions of the U.S. where the grid has low emissions associated with electricity output, one can reduce emissions by up to 92% when driving a BEV compared to an ICEV for cargo applications, as the extra energy needed to haul the heavier load has low associated emissions. In more carbon intensive grids, like Cincinnati, a BEV still has lower lifecycle emissions by up to 56% compared to an ICEV for cargo applications. PHEVs and HEVs for the same cargo load in Cincinnati have emissions which are 44–45% and 27% lower, respectively, than for ICEVs.

The impact of added weight on electric vehicle range is an important consideration (Figure 7). While added weight reduces range for both BEVs and ICEVs, the greater proportional loss in ICEVs has less impact on drivers due to their faster refueling times compared to BEV charging. The range of the BEV300 decreases by 111 miles (301 to 190 miles, 37% reduction) in a moderate climate like San Francisco, CA when 2500 lbs are added to the vehicle. It is also important to consider PHEVs which already have limited all-electric ranges when unloaded. In a moderate climate when carrying 2500 lbs of cargo, the all-electric ranges of the PHEV50 and PHEV35 are reduced to 31 and 22 miles, respectively. These low ranges limit the use of PHEVs in electric mode for cargo applications. City driving has significant benefits for EV range, increasing BEV300 range by 114 miles if no highway driving is performed with no cargo and 36 miles with 2500 lbs of cargo. Temperature also influences range, decreasing BEV300 annual average range by 35 miles in a cold city (Chicago, IL) compared to a moderate temperature city. In Chicago, similar to San Francisco, the range of the BEV300 decreases by 92 miles (266 to 174 miles, 35% reduction) when 2500 lbs are added to the vehicle. A BEV might be suitable for contractors with predictable short driving patterns but for contractors with unpredictable routes or long-distance driving requirements, PHEV or HEV options may be preferred. The reduced range of PHEVs would result in low utility factors, diminishing the benefits of electrification.

**Figure 7:**
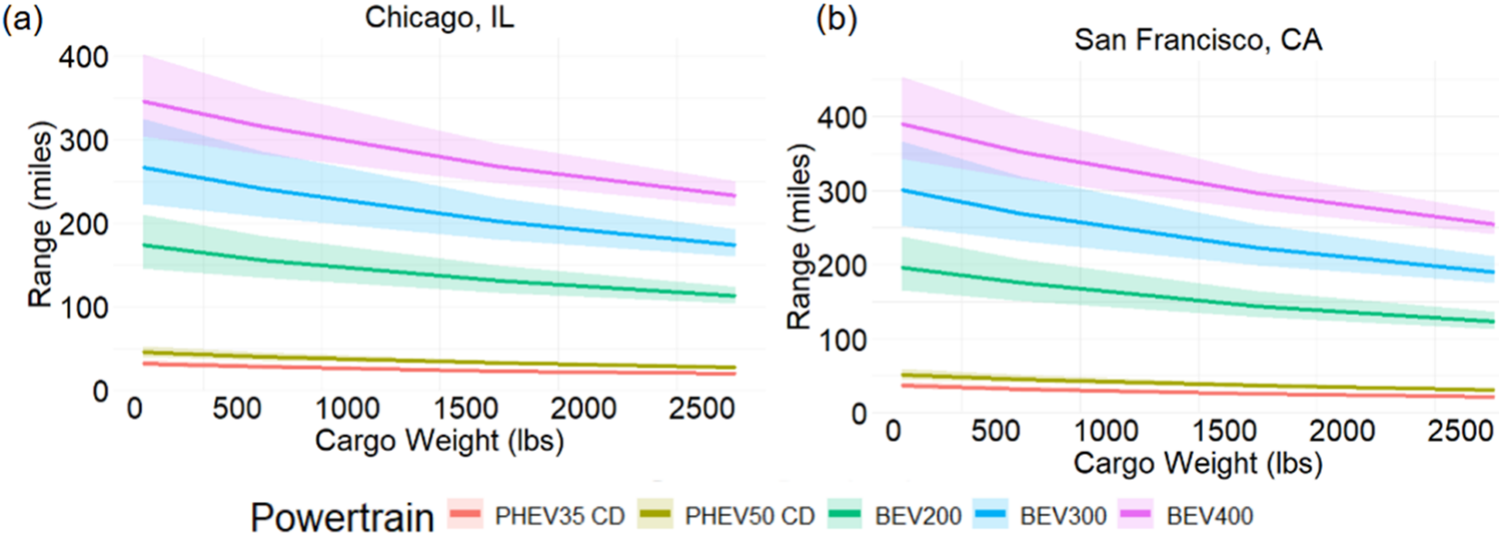
Effect of cargo on the all-electric range of pickup electric powertrains in (a) Chicago, IL and (b) San Francisco, CA. The shaded region shows the range from all highway driving (min range) to all city driving (max range) for each powertrain with the line representing a 43/57 city/highway split.

### 3.3 Regional and Consequential Lifecycle Emissions

When evaluating the emissions of electricity use, attributional LCA typically uses the average emissions factor of the electric grid (i.e., the total greenhouse gas emissions in a given region over a given time period divided by the total electricity generation or consumption in that same region and time period). Consequential analyses frequently use marginal emissions (i.e., the emissions per energy generation or demand of whichever generating resource increases its output in response to an incremental increase in electricity demand). Fossil generating assets are generally at the margin and hence consequential assessments generally equate to a more conservative estimate of the emissions benefit of technology adoption. This is appropriate when the change in electricity demand is small (e.g., the demand can be met by the marginal generator increasing output) but may not be as accurate when the change in demand is large (e.g., the demand requires new generators to be turned on, or for longer time scales, the demand induces the construction of new generators). To address this, NREL has developed long-run marginal emissions rates (LRMER), where changes in demand influence the long-term structure of the grid as well as the grid’s operation (see SI for details). EVs have a lifetime of about 15 years which is substantially longer than the about 5-year time scale for grid asset planning. When conducting a consequential assessment of EV adoption the use of LRMER is preferred.^43^

To conduct a consequential analysis we ran our model using the annual average short- and long-run marginal emission rates (SRMER and LRMER) for 2025–2040 from the 2023 Cambium Midcase scenario.^32^ The SRMERs are generally substantially lower than those prior to 2025 reported in the literature (see Figure S1).^41,42^ The grid and the loads on the grid have changed markedly over the past decade and are expected to continue to change with renewable generation increasing and additional loads from EVs, data centers, heat pumps, and industry.^32^

Figure 8 shows the benefits of electrification at a county level and illustrates the differences in lifecycle emissions across powertrains for the Midsize SUV using both attributional and consequential approaches. The figure shows the emissions difference between the powertrain in each column to the powertrain in each row. Regional variation has a minimal impact on comparisons of ICEV to HEV and PHEV to BEV. Apache County (Northeast Arizona) is an anomaly and results in much lower benefits when switching from an ICEV/HEV to a PHEV/BEV. Apart from this one county, regional variation can still account for ±150 g/mile in life cycle reductions of the BEV300 across the US. Parts of the Midwest and Appalachian regions tend to see the least benefit from electrification, whereas the northeast and Pacific Northwest tend to see the most benefit from electrification. Comparing an ICEV/HEV to a PHEV/BEV shows the highest GHG benefits relative to any other powertrain comparison across the country. BEVs and PHEVs provide similar benefits in the Midwest and Appalachian regions, but BEVs provide larger benefits than PHEVs outside of these areas. For reference, grid emissions rates vary by more than an order of magnitude, whereas fuel economy is only adjusted by a factor of 1.33 under the worst temperature conditions. Grid carbon intensity contributes more than temperature to regional variation in life cycle emissions. Notably, our analysis demonstrates increased electrification results in emissions reductions across all regions, a new finding that demonstrates the universal benefits of vehicle electrification across the continental United States. While Figure 8a–c uses different emissions metrics, regardless of the metric used (AER, LRMER or SRMER) electrified vehicles maintain their emissions advantage over ICEVs and HEVs across all U.S. counties.

**Figure 8:**
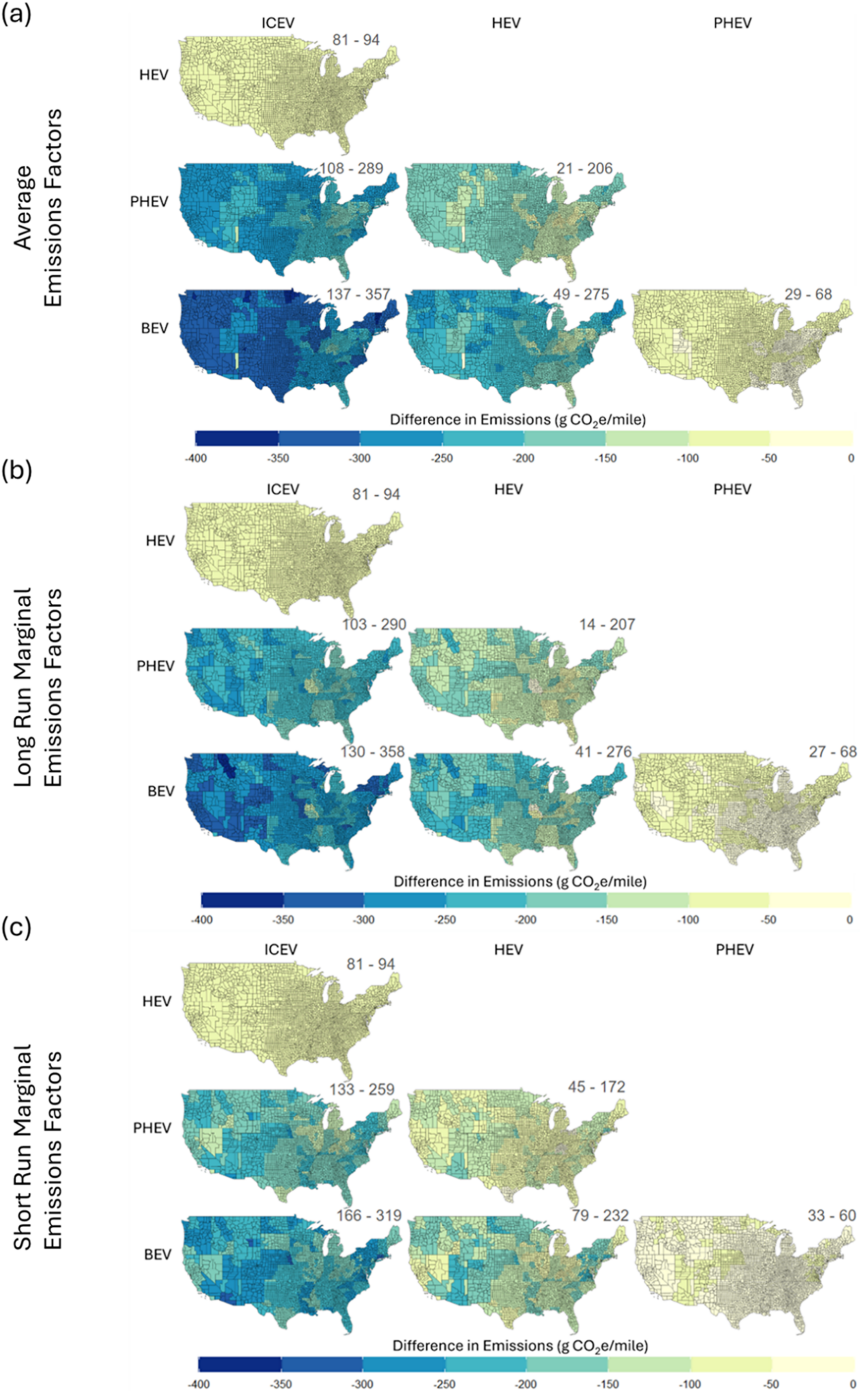
Lifecycle emissions (g CO_2_e/mile) benefits of increased electrification for a midsize SUV across the U.S using (a) average emissions factors (AEF), (b) long run marginal emissions factors (LRMEF) and (c) short run marginal emissions factors (SRMEF). Powertrains on the vertical axis are compared to those on the horizontal axis, e.g., left-hand column shows benefits of HEV, PHEV, and BEV compared to an ICEV. Spatial variation is explained by variation in electricity grid mix and average temperatures.

### 3.4 Sensitivity Analyses

#### 3.4.1 BEV Range

Lifecycle emissions of a 150-mile range BEV are 13–15% lower than the BEV300 across all vehicle classes. Of this decrease, about 78–87% is attributed to lower battery production emissions with the remainder coming from improved efficiency due to lower battery (and vehicle) weight. A 400-mile range BEV has 14–17% greater emissions than the BEV300 across all vehicle classes. Of this increase, about 69–73% is attributed to the increase in battery production emissions. Even with these increased emissions, the BEV400 still outperforms the PHEV35 and PHEV50 (under standard UF assumptions) with up to 30 and 25% lower lifecycle emissions, respectively (SI Figure S7).

#### 3.4.2 Vehicle Miles Traveled

We tested two scenarios for different VMT intensity based on the national highway travel survey.^30^ The high scenario considers a user with twice the average annual VMT. The low scenario considers a user with 25% lower annual VMT than average. The total lifecycle VMT for each vehicle stays constant (SI Figure S9).

The ICEV and HEV emissions per mile do not change as the emissions associated with fuel consumption remain constant over the life of the vehicle. The BEV emissions change because the grid emission intensity is higher in earlier years and lower later in the vehicle’s life. In the high scenario, the emissions per mile for the BEV increased by 16–19%. In the low scenario, emissions per mile decreased by 6% as a higher percentage of VMT is driven in future grids with lower grid intensity.

#### 3.4.3 Electricity Emissions Scenarios

Further analysis was performed to isolate the impact of the changing grid projections from the impact of improved vehicle efficiency. We compared the Cambium 2021 and 2023 Midcase scenarios, while holding all other variables constant. For the same 2025 MY vehicle, BEV300 lifecycle emissions were 17–33% lower for Cambium 2023 than Cambium 2021 projections, depending on vehicle class. Both models reflect policies in effect for their respective years; policies enacted between 2021 and 2023, along with cost and technology innovations, have led to a significant reduction in projected lifecycle emissions for electric vehicles.

Our analysis uses the Cambium Midcase scenario based on current policies; however, if the grid decarbonizes according to the goals of the Biden Administration (100% decarbonization by 2035), BEV emissions would be even lower. Using the Cambium 100% renewable electricity by 2035 scenario, BEV emissions are reduced by 12% (7g CO_2_e/mile) on average for the midsize sedan, with similar decreases at the county level (SI Note 9).

### 3.5 Comparison with Previous Work

There have been major changes in AERs recently (see SI) and we restrict our comparison to literature data for the U.S. evaluated with grid emission rates no older than 2020. For MY 2020 vehicles, Kelly et al. estimated BEV sedans had 166–209 g CO_2_e/mile.^40^ Woody et al. also investigated MY 2020 vehicles, and reported lower BEV sedan emissions of 141–182 gCO_2_e/mile when accounting for projected grid decarbonization over the lifetime of the vehicles.^13^ The lower emissions calculated in the present work for MY 2025 vehicles reflect the continued progress toward grid decarbonization and improved vehicle fuel efficiency, with the BEV sedan emissions of 88–113 g CO_2_e/mile. The number of locations in which ICEVs outperform BEVs has also been decreasing as the grid has decarbonized and grid projections have trended toward more rapid decarbonization. Woody et al. estimated that ICEVs had lower emissions than BEVs in 2.5–4.7% of counties, depending on the vehicle class.^13,37^ We find that for 2025 vehicles there are zero counties in which an ICEV has lower emissions than a comparable BEV. ICEV and BEV fuel consumptions are reduced by similar amounts (7–10 and 9–11%, respectively) from Woody et al. to this paper; therefore, this new finding is primarily due to lower projected grid emissions factors throughout the vehicle’s lifetime.

Kelly et al. estimated 219 g CO_2_e/mile for a MY 2020 PHEV50.^40^ We find that MY 2025 PHEV35 and PHEV50 have emissions of 160g CO_2_e/mile and 149 g CO_2_e/mile, respectively. The gap between PHEV emissions and BEV emissions has increased as the grid has decarbonized with a 35–71g CO_2_e/mile advantage for the BEV sedans in 2025 (and a larger gap for larger vehicle classes). Bruchon et al. reported a consequential assessment of the effects of an overnight replacement of 10% of the light-duty vehicle fleet with EVs in the PJM Interconnection area which has an electrical generation mix similar to the U.S. average in 2025.^45^ Two charging scenarios were considered; uncontrolled charging where the vehicle was charged on completion of the last trip of the day, or controlled charging where charging occurs at the lowest cost. For BEV300 sedans Bruchon et al. find a 39–43% (depending on charging scenario) reduction in emissions compared to ICEVs.^45^ Given the expectation that use of marginal instead of average emission rates will lead to decreased benefit for EV adoption this is qualitatively consistent with the 72% reduction we report in Figure 1. Reichmuth et al. conducted a cradle-to-grave attributional assessment for Power Control Areas and North American Reliability Regions in 2020 and reported that driving an average BEV results in lower emissions than driving an average ICEV everywhere in the U.S.^46^ However, Reichmuth et al. did not include the important effect of temperature on BEV efficiency and hence may have overestimated their benefits in some locations.^46^

### 3.6 Implications and Limitations

Improving our understanding of the key drivers of life cycle GHG emissions is crucial for reducing emissions and fostering more sustainable light duty transportation. National LDV lifecycle emissions range from 81–486 g CO_2_e/mile, offering up to 83% emissions reduction potential by electrifying and downsizing from an ICEV pickup to a BEV compact sedan. We estimate that powertrain electrification has greater potential for lifecycle emissions reduction relative to the benefits of downsizing alone. The complete array of options and emissions reductions is displayed in the 20 by 20 vehicle powertrain/class matrix (SI Figure S12). We have also developed a tool for drivers to calculate emissions for their location by county and use patterns (utility factor and drive cycle) that will be available online.

Regional variations in BEV emissions across the U.S. range from 34 to 341 g CO_2_e/mile depending on grid emissions, vehicle class, and battery size. In low-carbon grid areas like Valley County, MT, BEV200 pickup and compact sedan emissions are 90% and 93% lower than ICEV pickups. Even in Apache County, AZ, with the most carbon intensive grid, BEV200 pickup and compact sedan emissions are 40% and 62% lower than ICEV pickups. In clean grids, transitioning to BEVs or PHEVs driven >85% in electric mode offers the greatest emissions reductions compared to ICEVs or HEVs. In dirtier grids, BEVs consistently have lower lifecycle emissions than PHEVs, even at 100% UF. While a consequential analysis of the benefits of increased vehicle electrification shows lower emissions reductions than an attributional analysis, there remains a benefit in every county in the contiguous U.S.

We examine three user archetypes in this paper: commuter, long-distance traveler, and contractor. The commuter archetype, representing the most common regular trip types (work commutes, shopping/errands, and social/recreational activities) is particularly relevant for emissions reduction. While consumers have been trending toward larger vehicles over time,^47^ our results highlight the implications of consumer vehicle purchasing decisions. To aid in communicating results, we provide vehicle powertrain/class matrices in Figures 1 and 5, with a full enumeration of options in the SI, enabling consumers to make informed, emissions-conscious vehicle selections aligned with their actual usage patterns.

Utility factor and drive cycle are key use patterns for commuters. High UF PHEVs (UF > 75%) have emissions which are similar to those of BEVs and offer greater flexibility of operation. At standard UFs, PHEV50s have 58% lower GHG emissions than ICEVs while BEV300s have 72% lower GHG emissions than ICEVs.

For the contractor archetype, BEVs and PHEVs are more efficient than ICEVs when carrying cargo, resulting in lower additional emissions for the same added weight. However, cargo significantly impacts range; 2500 lbs reduces BEV and PHEV range by roughly one-third. This is especially important when considering the already limited all-electric ranges of PHEVs.

Our study has limitations that warrant further investigation. We did not examine charging times or frequency required for long-distance travel. While we used annual averages for emissions factors, the specific hours of the day in which charging takes places could result in higher or lower emissions estimates. It is difficult to predict how charge timing patterns may change with the widespread adoption of BEVs, especially if there are programs in place that incentivize charging during hours with lower emissions. Woody et al., highlighted the environmental implications of temporal variation in grid emissions and depth of discharge, showing that optimal charging decisions can notably reduce emissions.^13,48^ The long-run emissions rates from Cambium, which uses a constant load perturbation across all hours of the year, does not estimate how load timing may impact long-run consequential emissions. Factors such as cargo load and ambient temperature also affect charging times and frequency, potentially decreasing utility factors and increasing lifecycle emissions in practice. We did not examine other drive cycles such as aggressive driving, but the effect of aggressive driving on lifecycle emissions would be greater for gasoline powertrains compared to electric powertrains, as shown by Karabasoglu and Michalek.^49^ We acknowledge driving mileage varies by region,^50^ but we did not include this directly in the analysis. We instead address variations in mileage and UF as sensitivity cases. We also do not account for VMT variation based on changing refueling costs and convenience. Different regions also commonly have different road surface conditions such as the regularity of snow/ice coverage which can affect the fuel consumption and is not accounted for in our study. While our archetypes represent common use cases, they are not exhaustive. Future research should explore additional user profiles, considering factors like towing, variation in charging decisions, charging access disparities across demographic groups^51^ and regional vulnerabilities to electricity outages or extreme weather events.^52,53^ These considerations are important when comparing fully electric vehicles to less electrified options like PHEVs or HEVs. Expanding the range of archetypes and incorporating these factors would provide greater understanding of vehicle electrification’s real-world implications and guide more nuanced decision-making for diverse user groups. We also note that consumer vehicle choice is driven by many other factors such as cost, safety, and utility which are not addressed in our study. Electrification benefits from less costly and emissive driving may also induce more VMT demand.^54^ These rebound effects are not accounted for in our study. Other approaches to reducing GHG emissions such as using limited lithium supply to deploy a greater number of HEVs^55^ (compared to BEVs and PHEVs), are not considered in this study. As with other consequential assessments of EV adoption conducted to date, we have neglected the marginal consequences of decreased petroleum demand. This will likely cause an underestimate of EV benefits, as marginal crude oil has a higher upstream carbon intensity than average crude oil.^44^ We also use attributional results for the vehicle cycle, because there is a lack of data on marginal production emissions across the vehicle supply chain. Further work would be useful to better quantify these effects.

Consumers, automakers, and policy makers have key roles in reducing transportation sector emissions and meeting emissions reduction targets. Vehicle lifecycle emissions can be reduced by prioritizing BEVs where feasible, considering PHEVs with high utility factors, downsizing vehicles, evaluating regional grid conditions, and carefully considering different use cases.

## Supplementary Material



## Data Availability

Data will be made available upon request. The online tool developed for users to calculate emissions based on their vehicle choices can be found at the link below. https://css.umich.edu/research/projects/greenhouse-gas-reductions-driven-vehicle-electrification-across-powertrains.

## Notes

Comprehensive literature review, additional detailed methods, a detailed breakdown of vehicle cycle and total lifecycle greenhouse gas emissions, sensitivity analyses, and an extended 20 × 20 vehicle powertrain/class matrix supporting the main findings (PDF)
