# Supplementary material for: Greenhouse Gas Reductions Driven by Vehicle Electrification across Powertrains, Classes, Locations, and Use Patterns
Source: Environ Sci Technol. 2025 Aug 25;59(37):19768–80. doi: 10.1021/acs.est.5c05406 (PMC12461929; doi:10.1021/acs.est.5c05406)
Supplement: Supplementary file 1 [file es5c05406_si_001.pdf]

Supplemental Information

Greenhouse Gas Reductions Driven by Vehicle Electrification Across Powertrains, Classes, Locations, and Use Patterns

Elizabeth Smith[1,2], Maxwell Woody[2,3], Timothy J Wallington[1,2], Christian Hitt[1,2], Hyung Chul Kim[5], Alan I Taub[1,3,4], Gregory A Keoleian[1,2*]

[1] *Electric Vehicle Center, University of Michigan, Ann Arbor, MI 48109, United States of America*
[2] *Center for Sustainable Systems, School for Environment and Sustainability, University of Michigan, 440 Church Street, Ann Arbor, MI48019, United States of America*
[3]*Mechanical Engineering, University of Michigan, 2350 Hayward St Ann Arbor, MI 48109, United States of America*
[4]*Materials Science and Engineering, University of Michigan, 515 E. Jefferson St Ann Arbor, MI 48109, United States of America*
[5] *Research and Innovation Center, Ford Motor Company, Dearborn, MI 48121, United States of America*

*gregak@umich.edu

27 Pages
10 Figures
6 Tables

Introduction Supplement

Supplemental Note 1 – Lit. Review and Comparison with Real Vehicles

Methods Supplement

Supplemental Note 2 – Vehicle Assumptions

Supplemental Note 3 – Typical Drive Pattern

Supplemental Note 4 – Drive Cycle Adjustment Methods

Supplemental Note 5 – Regional Variation Methods

Supplemental Note 6 – Fuel Reduction Values

Results Supplement

Supplemental Note 7 – National Average Lifecycle Emissions

Sensitivity Supplement

Supplemental Note 8 – BEV Battery Size

Supplemental Note 9 – Electricity Emissions Factors

Supplemental Note 10 – Vehicle Miles Travelled

Vehicle Decision Matrix

Supplemental Note 11 – Vehicle Decision Matrix

Supplemental References

Supplemental Note 1a – Previous life cycle assessment studies of EVs

Table S1. Literature Review

**Study**	**Vehicle type**	**Powertrain**	**Production Phase Included**	**Operation Emissions**			**Regional Temperature variation**	**Use Cases**		
				**Emissions Rates**	**Data Timeframe**	**Regional Variation**		**Utility Factor**	**Drive Cycle**	**Cargo**
Samaras and Meisterling, 2008[1]	Sedan	ICEV, HEV, PHEV	Yes	AER*	Historic	United States	–	Dictated by battery size and NHTS daily travel distance data	EPA Fuel Economy Labels	N/A
Stephen and Sullivan, 2008[2]	LDV	ICEV, HEV, PHEV	No	AER, MER	Historic, Projection	NERC Regions	–	100%	European Fuel Cell Forum	N/A
Jaramillo et al., 2009[3]	Sedan	ICEV, HEV, PHEV, FCV	Yes	AER*	Historic	United States (National)	–	Dictated by battery size and NHTS daily travel distance data	Fuel Economies from a variety of sources (EPRI, EPA, Shiau et al.)	N/A
Faria et al., 2013[4]	Sedan	ICEV, PHEV, BEV	Yes	AER	Historic, Projection	Poland, Portugal, and France (National)	–	80%	Manufacturer Data	N/A
Graff Zivin et al., 2014[5]	Sedan	ICEV, HEV, BEV	No	MER	Historic	NERC Region	-	–	Manufacturer Data, 2012 fleet average	NA
Lewis, 2014[6]	Sedan	ICEV, HEV, PHEV	Yes	AER	Historic	–	–	SAE	Autonomie Simulation	NA
Onat et al., 2015[7]	Sedan	ICEV, HEV, PHEV, BEV	Yes	AER, MER	Historic, Projection	NERC Regions	–	State based UF for set of AER	EPA Fuel Economy Labels	NA
Tamayao et al., 2015[8]	Sedan	ICEV, HEV, PHEV, BEV	Yes	AER, MER	Historic	NERC Regions	–	US NHTS 2009 national Distribution	EPA Combined	NA

Orsi et al., 2016[9]	Sedan	ICEV, HEV, PHEV, BEV, CNG	-	AER	Historic	Brazil, China, France, Italy, and the United States	-	6 drive cycles: ARTEMIS Urban, Japan 08, US06, ARTEMIS Motorway, FTP, SC03		NA
Holland et al. 2022[10]	Sedan	ICEV, BEV	No	SRMER	Historic	NERC Regions	Yes	-	EPA Labels	NA
Hoehne et al., 2016[11]	Sedan	PHEV, BEV	–	MER	Historic	NERC Regions	–	All electric, Select charging scenarios	Urban driving	NA
McLaren, 2016[12]	Sedan	ICEV, PHEV, BEV	–	AER	Theoretic	–	–	Driving Scenario output by BLAST-V		NA
Yuksel, 2016[13]	Sedan	ICEV, HEV, PHEV, BEV	Yes	MER	Historic	NERC Regions	Yes	NHTS(2009) state distribution	EPA city, highway or combined based on county urbanization level	NA
Lombardi et al., 2017[14]	Midsize car	ICEV, PHEV, BEV, Fuel Cell Hybrid	Yes	AER	Historic	Italy, United States, France	–	100% UF	55/45 highway/city split	N/A
Van Mierlo, 2017[15]	Sedan	HEV, PHEV, CNG, BG	Yes	AER	Historic	Belgium	–	Not Stated	Not Stated	N/A
Bicer and Dincer, 2018[16]	Sedan	ICEV, PHEV, BEV, CNG	Yes	AER	Historic, Theoretic	Europe	–		50%	
De Souza, 2018[17]	Sedan	ICEV, PHEV, BEV	Yes	AER	Historic	Brazil	–	62% UF	Brazilian Standard	NA
Elgowainy, 2018[18]	Sedan	ICEV, HEV, PHEV, BEV, FCEV, CNG	Yes	AER	Historic	–	–	SAE	combined	NA
Karaaslan, 2018[19]	SUV	ICEV, PHEV, BEV, FCEV	Yes	AER	Historic	–	–	54% UF	Manufacturer Data	NA

Kawamoto et al. 2019[20]	Sedan	ICEV, BEV	Yes	AER	Historic	EU, Japan, U.S, China	No	-	US 5 cycle, Europe NEDC, Japan JC0, China NEDC, Australia NEDC	NA
Desai et al. 2019[21]	Sedan, SUV, Minivan, Pickup truck	ICEV, HEV, PHEV, BEV	Yes	SRMER	Historic	U.S States	No		EPA Fuel Economy Labels	No
Gai et al. 2019[22]	Sedan, Pickup truck	ICEV, BEV	Yes	SRMER	Historic	Toronto, Canada	No	-	EPA Fuel Economy Labels	No
D. Wu, 2019[23]	Sedan	ICEV, HEV, PHEV, BEV	Yes	AER	Historic	eGRID regions	Yes	SAE	combined	
Tong and Azevedo, 2020[24]	Sedan, SUV, Transit bus	ICEV, HEV, BEV	Yes	SRMER	Historic	U.S. counties	No	-	GREET	No
Gan et al., 2021[25]	Sedan	ICEV, HEV, PHEV, BEV	–	AER	Historic	Chinese Provinces	Yes	SAE	Combined	NA
Settey et. al., 2021[26]	Small Commercial Vehicle	BEV	–	—	Historic	–	–	–	Mixed Profile (speeds, incline, etc.)	Yes
Woody et al., 2022[27]	Sedan, SUV, Pickup	ICEV, HEV, BEV	Yes	AER, MER, LRMER	Projection	US States	Yes	–	County based distribution	NA
Reichmuth et al., 2022[28]	Sedan, Pickup truck	ICEV, BEV	Yes	AER	Historic	United States, Power control areas	No	-	Greet	No
Kelly et. al, 2022[29]	Sedan, SUV	ICEV, HEV, PHEV, BEV	Yes	AER	Historic and Projection	U.S	No	-	GREET	No
Rashid and Pagone,		HEV, PHEV	Yes	AER	Historic	National	–	GREET	GREET	NA

2023[30]										
Jenn et al., 2023[31]	--	ICEV, BEV	No	SRMER	Projection	California	No	-	-	NA
Bruchon et al., 2024[32]	Sedan	ICEV, HEV, PHEV, BEV	Yes	SRMER	Projection	PJM	No	GREET	GREET	NA
Singh et al., 2024[33]	Sedan	HEV, BEV	Yes	SRMER, AER	Historic	NERC regions	Yes	-	UDDS, HWFET	NA
Maselli et al., 2025[34]	Sedan	ICEV, BEV	Yes	AER	Historic	Sao Paulo, Brazil	No	-	EPA label, eco-driving, aggressive driving	NA
This Study	Sedan, SUV, Pickup	ICEV, HEV, PHEV, BEV	Yes	AER, SRMER LRMER	Projection	Balancing Areas	U.S Counties	All	All	Yes

* Included "high carbon" and "low carbon" scenarios to simulate possibilities for marginal load.

As illustrated by the list in Table S1, numerous lifecycle assessments of the GHG emissions benefits of EVs have been published. Most studies have employed attributional methods using average emission rates (AER). Some studies have employed consequential using marginal emission rates. Marginal emissions can be estimated over the short-run or long-run. In the Cambium model, short-run marginal emission rates (SRMER) are defined as the emissions per unit change in electricity consumption, where the structure of the electrical grid (e.g., the generation, transmission, and distribution assets) is fixed.[35] Long-run marginal emission rates (LRMER) are defined as the emissions per unit change in electricity consumption, where the influence of the change in demand on both the operation and structure of the grid is taken into account (i.e., structure of grid is not fixed). The LRMER in Cambium is calculated by perturbing the total demand by 5%. The LRMERs from Cambium would not be valid to evaluate scenarios where EV charging results in a greater than 5% increase in total load on the electrical system at any point in the day. The LRMER concept is relatively new and was introduced in 2014 by Hawkes[36]. Guidance on the use of LRMER has been provided by Gagnon et al. who conclude "it is likely that interventions lasting 5 years or longer would be sufficient to warrant using long-run metrics for most locations in the US, given the duration of most planning cycles".[35] The lifetime of vehicles is approximately 15 years and hence it is clear that consequential life assessments need to include consideration of LRMER.

Table S1 highlights the key factors included in published lifecycle assessments. It can be difficult to make detailed comparisons of the results reported in different studies because there are substantial differences in the assumptions and scopes of the different studies. Key factors that differ across the published studies include the vehicle classes and vehicle powertrains considered, whether emissions from the vehicle cycle

are included, whether the effect of ambient temperature is included, and the geographic and temporal focus. The EV charging schedule is also an important factor with different assumptions in different studies.

The increasing availability of renewable energy (wind and solar) together with a switch away from coal to gas has led to substantial reductions in emissions associated with electricity generation in the U.S. Figure S1 shows AER, SRMER, and LRMER from the Cambium model, Holland et al., the EPA eGRID model and GREET.[10,37–39] There has been a marked decline in AER from 2010 to 2020 and this trend in projected to continue. There was little or no trend in SRMER in 2010-2020, but a decline is projected over the next 10-20 years. The historic SRMERs reflect a mixture of coal plants and natural gas plants as the marginal generators, and in the Cambium 2023 projections there is a significant reduction in coal generation between 2025 and 2030. The Cambium SRMEFs reach ~400 g CO2e/kWh in 2035-2050, reflecting a grid in which coal generators are mostly phased out, resulting in natural gas being the predominant marginal generator. As with SRMER, a substantial decline in LRMER is projected in the future. With the major decreases in emissions rates the relevance for current and future introduction of EVs of LCAs conducted using historic emission rates is questionable.

The focus of the present study is a vehicle entering service in 2025 with emissions calculated over the lifetime of the vehicle (through 2040). In the comparison of our results with literature studies in section 3.5 of the manuscript we restrict our comparison to literature data using grid emission rates no older than 2020.

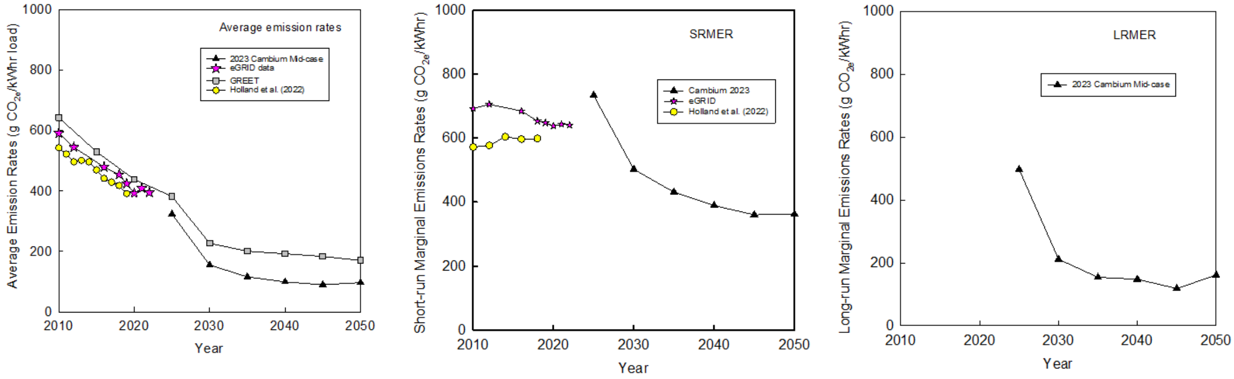

Figure S1. AER, SRMER, and LRMER from Cambium, eGRID, GREET, and Holland et al.[10] for 2010-2050.

Supplemental Note 1b - Comparison with Real Vehicles

Table S2. Real World Vehicle Data from FuelEconomy.gov

Vehicle Powertrain	Lab Year	Vehicle Class	Name of Vehicle	City	Highway	MPGe 43-57	Wh/mi 43-57	Electric Range (mi)	Test Weight (lbs)
BEV	2024	Pickup	Chevrolet Silverado EV	72	62	66	508		9000
BEV	2024	Pickup	Ford F-150 Lightning 4WD Extended Range	87	63	73	460	321	7000
BEV	2024	Pickup	Ford F-150 Lightning 4WD	76	61	67	500		6500
BEV	2024	Pickup	GMC Hummer EV Pickup	59	48	53	639		8500
BEV	2024	Pickup	Rivian R1T	74	66	69	485	315	7000
BEV	2024	Sedan	Hyundai Ioniq 6 Long range RWD (18 inch Wheels)	153	127	138	244		4500
BEV	2024	Sedan	Hyundai Ioniq 6 Long range AWD (18 inch Wheels)	130	111	119	283		4500
BEV	2024	Sedan	MINI Cooper SE Hardtop 2 door	119	100	108	312		3500
BEV	2024	Sedan	Nissan LEAF	123	99	109	308		3875
BEV	2024	Sedan	Nissan LEAF SV	121	98	108	312		4250
BEV	2024	SUV	Subaru Solterra AWD	114	94	103	329		4750
BEV	2024	SUV	Audi Q4 40 e-tron	112	94	102	331		5250
BEV	2024	SUV	Audi Q8 e-tron quattro	80	83	82	412		6000
BEV	2024	SUV	BMW iX xDrive40 (20 inch Wheels)	87	85	86	393		5500
BEV	2024	SUV	Cadillac LYRIQ	95	82	88	385		6000
BEV	2024	SUV	Chevrolet Blazer EV AWD	103	88	94	357		5500
BEV	2024	SUV	Genesis Electrified GV70	98	93	95	354		5250
BEV	2024	SUV	GMC Hummer EV SUV	59	48	53	639		9000
BEV	2024	SUV	Hyundai Ioniq 5 Long range AWD	110	88	97	346		4750
BEV	2024	SUV	Kia EV6 AWD GT	88	77	82	412		5000
BEV	2024	SUV	Kia Niro Electric	126	101	112	302		4000
BEV	2024	SUV	Mercedes-Benz EQS 680 4matic Maybach (SUV)	75	77	76	443		7000
BEV	2024	SUV	Volkswagen ID.4 AWD Pro	108	96	101	333		5250
BEV	2024	SUV	Volvo C40 Recharge	118	96	105	320		4750

HEV	2024	Sedan	2024 Hyundai Elantra Hybrid Blue	51	58	55			3375
HEV	2024	Sedan	2024 Toyota Corolla Hybrid	53	46	49			3750
HEV	2024	Sedan	2024 Hyundai Elantra Hybrid	49	52	51			3375
HEV	2024	Sedan	2024 Audi A5 Coupe quattro	24	32	29			4000
HEV	2024	Sedan	2024 Toyota Prius	57	56	56			3375
HEV	2024	Sedan	2024 Toyota Camry Hybrid LE	51	53	52			3750
HEV	2024	Sedan	2024 Honda Accord Hybrid	51	44	47			3625
HEV	2024	Sedan	2024 Hyundai Sonata Hybrid	44	51	48			3875
HEV	2024	SUV	2024 Kia Niro FE	53	54	54			3375
HEV	2024	SUV	2024 Kia Sportage Hybrid FWD	42	44	43			4000
HEV	2024	SUV	2024 Lexus UX 250h	43	41	42			3875
HEV	2024	SUV	2024 Honda CR-V FWD	43	36	39			3875
HEV	2024	SUV	2024 Ford Escape FWD HEV	42	36	39			4000
HEV	2024	SUV	2024 Toyota RAV4 Hybrid AWD	41	38	39			4000
HEV	2024	SUV	2024 Hyundai Tucson Hybrid Blue	40	37	38			4000
HEV	2024	SUV	2024 Toyota Highlander Hybrid	36	35	35			4750
HEV	2024	SUV	2024 Lincoln Nautilus HEV AWD	30	31	31			5000
HEV	2024	SUV	2024 Mercedes-Benz GLA250	26	33	30			
HEV	2024	SUV	2024 Lexus TX 500h AWD	27	28	28			5250
HEV	2024	SUV	2024 Mazda CX-90 4WD	24	28	26			5250
HEV	2024	SUV	2024 Land Rover Range Rover P400 LWB MHEV	18	24	21			5000
HEV	2024	Pickup	2024 Ford Maverick HEV FWD	42	33	37			4000
HEV	2024	Pickup	2024 Ford F150 Pickup 4WD HEV	22	24	23			6000

HEV	2024	Pickup	2024 Ram 1500 HFE 2WD	20	26	23			5000
ICEV	2024	Pickup	Chevrolet Colorado 2WD	19	24	22			4750
ICEV	2024	Pickup	Chevrolet Silverado 2WD	18	22	20			5500
ICEV	2024	Pickup	Ford Maverick AWD	22	29	26			4000
ICEV	2024	Pickup	Ford Ranger 2WD	21	25	23			4500
ICEV	2024	Pickup	GMC Canyon 2WD	19	23	21			5000
ICEV	2024	Pickup	GMC Sierra 4WD	17	20	19			5500
ICEV	2024	Pickup	Nissan Frontier 2WD	18	24	21			4750
ICEV	2032	Pickup	Toyota Tundra 2WD	18	23	21			6000
ICEV	2024	Sedan	Mazda 3 5-Door 2WD	26	36	32			3750
ICEV	2024	Sedan	MINI Cooper Hardtop 4 door	27	38	33			3250
ICEV	2024	Sedan	Honda Accord	29	37	34			3625
ICEV	2024	Sedan	Honda Civic 4Dr 1	33	42	38			3125
ICEV	2024	Sedan	Honda Civic 4Dr 2	31	40	36			3250
ICEV	2024	Sedan	Honda Civic 5Dr	31	39	36			3375
ICEV	2024	Sedan	Hyundai Elantra 2.0 L, 4 cyl, Automatic (AV-S1)1	32	41	37			3375
ICEV	2024	Sedan	Hyundai Elantra 2.0 L, 4 cyl, Automatic (AV-S1)2	31	40	36			3375
ICEV	2024	Sedan	Kia Forte	22	31	27			3375
ICEV	2024	Sedan	Kia Forte FE	30	41	36			
ICEV	2024	Sedan	Kia K5	27	37	33			3750
ICEV	2024	Sedan	Mitsubishi Mirage G4	35	41	38			2500
ICEV	2024	Sedan	Mitsubishi Mirage	36	43	40			2375
ICEV	2024	Sedan	Nissan Altima SR	25	34	30			3625
ICEV	2024	Sedan	Nissan Kicks	31	36	34			3000
ICEV	2024	Sedan	Nissan Sentra	30	40	36			3375
ICEV	2024	Sedan	Nissan Versa	32	40	37			2875
ICEV	2024	Sedan	Subaru Impreza	27	34	31			3500
ICEV	2024	Sedan	Subaru Legacy AWD	23	31	28			3875
ICEV	2024	Sedan	Toyota Camry XLE/XSE	27	38	33			3625
ICEV	2024	Sedan	Toyota Corolla Hatchback	32	41	37			3375
ICEV	2024	Sedan	Toyota Corolla	32	41	37			3375
ICEV	2024	Sedan	Volkswagen GTI	24	33	29			3500
ICEV	2024	Sedan	Volkswagen Jetta	30	41	36			3250
ICEV	2024	SUV	Buick Enclave AWD	17	25	22			5000
ICEV	2024	SUV	Chevrolet Suburban 2WD	15	20	18			6500

ICEV	2024	SUV	GMC Yukon 2WD	15	20	18			6500
ICEV	2024	SUV	Hyundai Palisade FWD	19	26	23			4500
ICEV	2024	SUV	Volkswagen Atlas SE 4motion	19	26	23			4750
ICEV	2024	SUV	Audi Q3 quattro	22	29	26			4500
ICEV	2024	SUV	Audi SQ5	19	24	22			4750
ICEV	2024	SUV	Buick Encore GX AWD	26	28	27			3500
ICEV	2024	SUV	Buick Envista	28	32	30			3375
ICEV	2024	SUV	Cadillac XT5 AWD	18	26	23			4750
ICEV	2024	SUV	Chevrolet Blazer AWD	22	27	25			4500
ICEV	2024	SUV	Chevrolet Equinox AWD	24	30	27			3875
ICEV	2024	SUV	Chevrolet Tahoe 2WD	15	20	18			6000
ICEV	2024	SUV	Chevrolet Trailblazer AWD	26	29	28			3500
ICEV	2024	SUV	Chevrolet Trax	28	32	30			3250
ICEV	2024	SUV	Dodge Durango AWD	18	25	22			5500
ICEV	2024	SUV	Dodge Hornet AWD	21	29	26			4000
ICEV	2024	SUV	Ford Bronco Sport 4WD	25	29	27			4000
ICEV	2024	SUV	Ford Edge AWD	21	28	25			5000
ICEV	2024	SUV	Ford Expedition 2WD	17	23	20			6000
ICEV	2024	SUV	Ford Expedition Timberline AWD	16	19	18			6000
ICEV	2024	SUV	GMC Terrain AWD	23	28	26			4000
ICEV	2024	SUV	Honda CR-V FWD	28	34	31			3750
ICEV	2024	SUV	Honda Passport AWD	19	24	22			4500
ICEV	2024	SUV	Honda Pilot AWD	19	25	22			4750
ICEV	2024	SUV	Hyundai Kona AWD	27	29	28			3250
ICEV	2024	SUV	Hyundai Tucson AWD	23	29	26			3750
ICEV	2024	SUV	Jeep Compass 4WD	24	32	29			3875
ICEV	2024	SUV	Jeep Wagoneer 2WD	17	24	21			6000
ICEV	2024	SUV	Jeep Wrangler 2dr 4WD	20	21	21			4750
ICEV	2024	SUV	Kia Seltos FWD	28	34	31			3250
ICEV	2024	SUV	Kia Telluride AWD	18	24	21			4750
ICEV	2024	SUV	Mazda CX-30 4WD	22	30	27			3875
ICEV	2024	SUV	Mitsubishi Eclipse Cross 2WD	25	28	27			3750
ICEV	2024	SUV	Mitsubishi Outlander 4WD	24	30	27			4250
ICEV	2024	SUV	Nissan Murano FWD	20	28	25			4250

ICEV	2024	SUV	Nissan Pathfinder 4WD	21	27	24			4750
ICEV	2024	SUV	Subaru Ascent	20	26	23			5000
ICEV	2024	SUV	Subaru Crosstrek AWD	27	34	31			3750
ICEV	2024	SUV	Subaru Forester Wilderness AWD	25	28	27			4000
ICEV	2024	SUV	Subaru Outback AWD	26	32	29			4250
ICEV	2024	SUV	Toyota 4Runner 2WD	16	19	18			4750
ICEV	2024	SUV	Toyota Grand Highlander AWD LE/XLE	21	27	24			4750
ICEV	2024	SUV	Toyota RAV4	27	35	32			3750
ICEV	2024	SUV	Volkswagen Tiguan	23	30	27			4250
PHEV	2023	Sedan	MINI Cooper SE Countryman All4			29	460	18	
PHEV	2024	Sedan	Toyota Prius Prime SE			52	260	45	3750
PHEV	2024	Sedan	Toyota Prius Prime SE			52	260	45	3750
PHEV	2024	Sedan	Toyota Prius Prime			49	300	40	3875
PHEV	2023	Sedan	Toyota Prius Prime			49	300	40	3875
PHEV	2024	SUV	Kia Sorento Plug-in Hybrid			34	420	32	4750
PHEV	2024	SUV	Lexus NX 450h Plus AWD			36	400	38	4750
PHEV	2024	SUV	Alfa Romeo Tonale eAWD			29	440	33	
PHEV	2024	SUV	BMW XM			14	730	31	6500
PHEV	2024	SUV	Dodge Hornet PHEV AWD			29	440	33	4500
PHEV	2024	SUV	Hyundai Tucson Plug-in Hybrid			35	420	33	4500
PHEV	2024	SUV	Jeep Grand Cherokee 4xe			23	580	26	5500
PHEV	2024	SUV	Jeep Wrangler 4dr 4xe			20	680	22	5500
PHEV	2024	SUV	Kia Sportage Plug-in Hybrid			35	400	34	4500
PHEV	2024	SUV	Mitsubishi Outlander PHEV			26	520	38	5000
PHEV	2024	SUV	Toyota RAV4 Prime 4WD			38	350	42	4500
PHEV	2024	SUV	Volvo XC60 T8 AWD Recharge			28	500	35	5000
PHEV	2024	SUV	Volvo XC90 T8 AWD Recharge			27	550	32	5500

PHEV	2024	SUV	Dodge Hornet PHEV AWD			29	440	33	4500
PHEV	2024	SUV	Kia Sorento PHEV			34	420	32	4750

Table S3. Autonomie Vehicle Data

Vehicle Powertrain	Lab Year	Vehicle Class	MPGe 43-57	Wh/mi 43-57	Electric Range (mi)	Test Weight (lbs)
BEV	2025	Pickup		331.8	150	4839
BEV	2025	Pickup		333.3	200	4993
BEV	2025	Pickup		344.4	300	5359
BEV	2025	Pickup		375.3	400	5789
BEV	2025	Sedan		191.5	150	3025
BEV	2025	Sedan		192.5	200	3122
BEV	2025	Sedan		202.1	300	3329
BEV	2025	Sedan		221.9	400	3633
BEV	2025	Sedan		204.5	150	3461
BEV	2025	Sedan		205.5	200	3560
BEV	2025	Sedan		215.5	300	3774
BEV	2025	Sedan		235.9	400	4081
BEV	2025	SUV		265.1	150	3920
BEV	2025	SUV		273.2	200	4054
BEV	2025	SUV		283.7	300	4354
BEV	2025	SUV		305.3	400	4713
BEV	2025	SUV		240.1	150	3662
BEV	2025	SUV		248.4	200	3805
BEV	2025	SUV		258.2	300	4050
BEV	2025	SUV		279.8	400	4405
HEV	2025	Pickup	32			5086
HEV	2025	Sedan	48			3351
HEV	2025	Sedan	44			3843
HEV	2025	SUV	38			4178
HEV	2025	SUV	40			3990
ICEV	2025	Pickup	24			5013
ICEV	2025	Sedan	37			3188
ICEV	2025	Sedan	34			3710
ICEV	2025	SUV	29			4134
ICEV	2025	SUV	31			3858
PHEV	2025	Pickup	37	385.8		5247
PHEV	2025	Pickup	37	395.6		5368
PHEV	2025	Sedan	53	256		3419
PHEV	2025	Sedan	52	261.6		3499
PHEV	2025	Sedan	51	267.6		3922
PHEV	2025	Sedan	50	276.2		3999
PHEV	2025	SUV	42	337.1		4299
PHEV	2025	SUV	43	338.4		4403

PHEV	2025	SUV	45	315.8		4083
PHEV	2025	SUV	45	313.3		4178

Table S4. Vehicle Characteristics from the Autonomie Model.[40]

Vehicle Type	Powertrain	Curb Weight (lbs)	Vehicle Battery Size (kWh)	Fuel Economy (MPG)	Fuel Economy (Wh/mi)
Compact Sedan	ICEV	2,888	--	37	--
	HEV	3,051	1	48	--
	PHEV 35	3,120	12	53	290
	PHEV 50	3,199	17	52	297
	BEV 200	2,822	41	--	218
	BEV 300	3,029	64	--	229
Midsize Sedan	ICEV	3,411	--	34	--
	HEV	3,543	1	44	--
	PHEV 35	3,622	12	51	303
	PHEV 50	3,699	18	50	313
	BEV 200	3,261	43	--	233
	BEV 300	3,474	67	--	244
Small SUV	ICEV	3,558	--	31	--
	HEV	3,691	1	40	--
	PHEV 35	3,783	14	45	358
	PHEV 50	3,878	20	45	355
	BEV 200	3,505	53	--	282
	BEV 300	3,750	81	--	293
Midsize SUV	ICEV	3,834	--	29	--
	HEV	3,878	1	38	--
	PHEV 35	3,999	15	42	382
	PHEV 50	4,103	22	43	384
	BEV 200	3,754	57	--	310
	BEV 300	4,054	90	--	322
Pickup	ICEV	4,713	--	24	--
	HEV	4,786	1	32	--
	PHEV 35	4,947	18	37	437
	PHEV 50	5,068	26	37	449
	BEV 200	4,694	69	--	378
	BEV 300	5,060	110	--	390

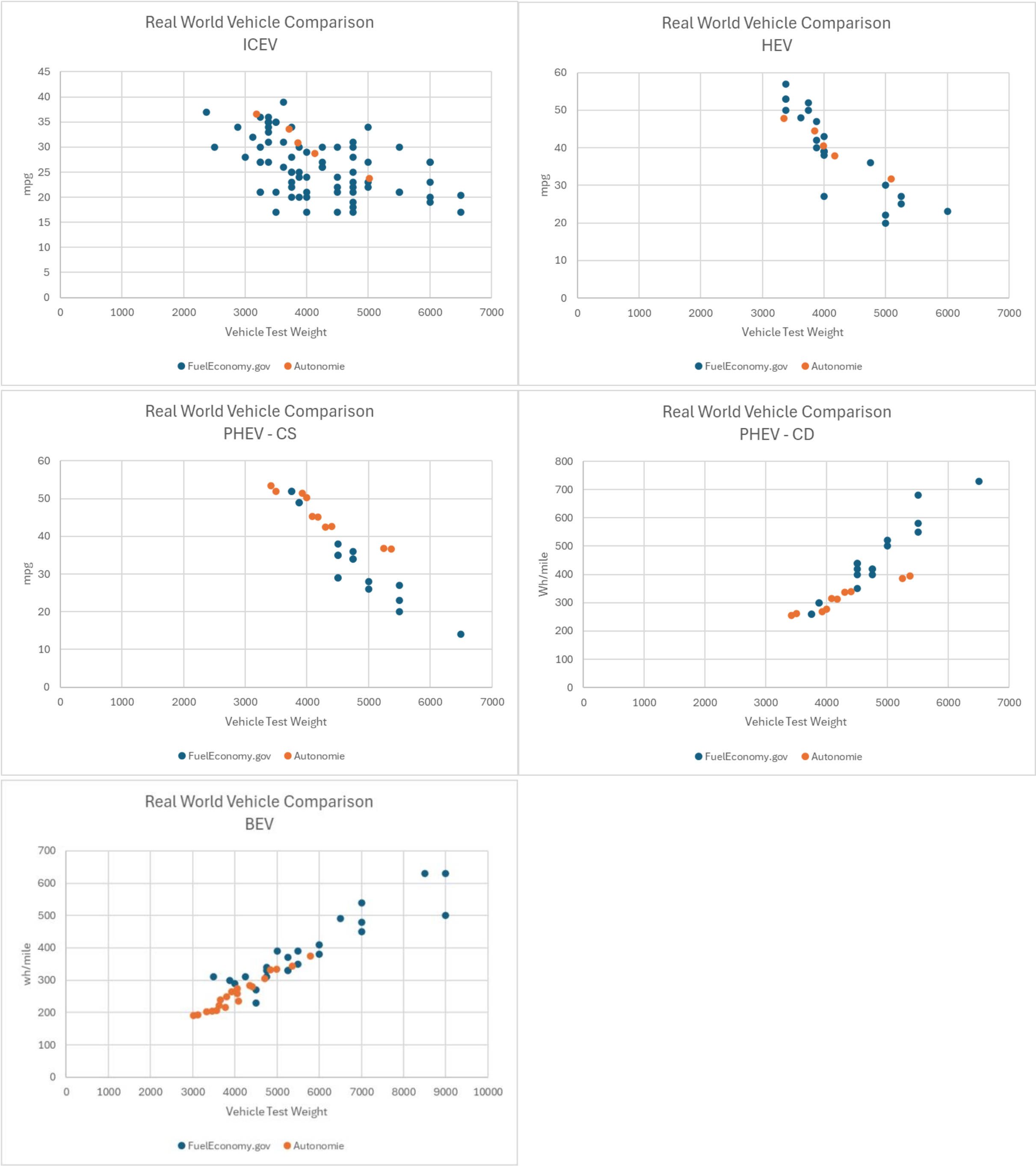

Figure S2. Autonomie vehicle parameters compared with real vehicle data.[41]

Supplemental Note 2 – Vehicle Assumptions
Vehicle parameters were obtained from the Argonne National Laboratory Autonomie model for 2025 model year conventional ICEV, Par HEV SI, Par PHEV35, Par PHEV50, BEV150, BEV200, BEV300 and BEV400 options.[40]
The BEVs have a battery energy density of 244 Wh/kg and the PHEVs have a battery energy density of 153 Wh/kg. We used this data to calculate the vehicle cycle emissions using GREET including vehicle components (body, powertrain, transmission, chassis, motor, generator, other electronic components), fluids used over the lifetime of the vehicle, vehicle batteries (lead-acid for ICEV and Li-ion (NMC111) for PHEV and BEV vehicles), and finally, assembly and disposal of the vehicle. Li-ion battery recycling is not included in this analysis.

Supplemental Note 3 - Typical Drive Pattern
We assume typical driving: a city/highway split of 43/57, SAE standard utility factors for PHEV defined by battery size (58% for PHEV35, 69% for PHEV50),[42] a set VMT schedule, and no cargo. The lifetime VMT for each vehicle class is given in Table S2 and the annual VMT schedule in Figure S2.

Table S5. Vehicle lifetime in years and miles, from NHTS [43]

Vehicle Type	Lifetime VMT (miles)	Lifetime Years (years)
Sedan	191,386	14.85
SUV	211,197	15.94
Pickup	244,179	18.61

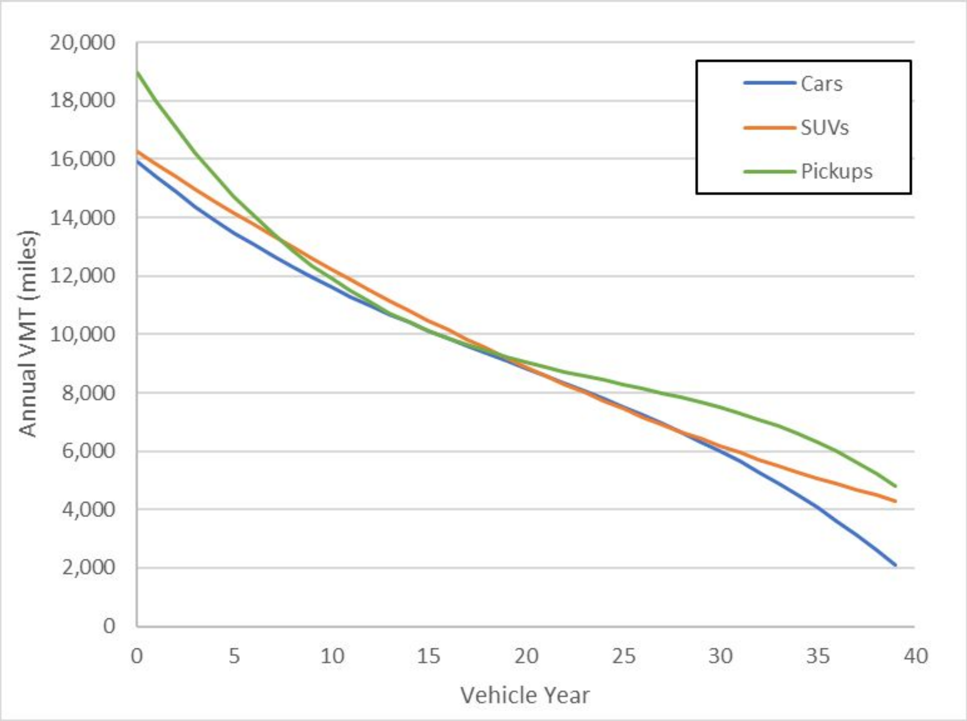

Figure S3. Annual VMT of each vehicle class (sedans, SUVs and pickups) by vehicle age[43]

Supplemental Note 4 – Drive Cycle Adjustment Methods

To account for different driving conditions, the fuel economy of each vehicle is adjusted accordingly. The unadjusted fuel economies for Urban Dynamometer Driving Schedule (UDDS) and the Highway Fuel Economy Test (HWFET) are used to represent city and highway driving, respectively. However, since these two-cycle tests do not fully capture real-world driving behavior—such as higher acceleration and speed, use of heating and AC, and varying road conditions—we applied adjustments recommended by the U.S. EPA[41], as utilized by Elgowainy et al.[44]

For ICEVs, HEVs, and PHEVs in charge sustaining (CS) mode, the following equations were used with fuel economy in miles/gallon for on-road (OR) adjustments:

$$FE_{g,city}^{OR} = \frac{1}{0.003259 + (\frac{1.1805}{FE_{UDDS}})}$$

$$FE_{g,Highway}^{OR} = \frac{1}{0.001376 + (\frac{1.3466}{FE_{HWFET}})}$$

For BEVs and PHEVs in charge depleting (CD) mode, the following equations were used with fuel economy in Wh/mile for on-road (OR) adjustments:

$$FE_{e,city}^{OR} = \frac{FE_{UDDS}}{0.7}$$

$$FE_{e,Highway}^{OR} = \frac{FE_{HWFET}}{0.7}$$

Supplemental Note 5 – Regional Variation Methods

Three key factors are considered to account for the differences in vehicle operating emissions across the U.S.: variability in driving cycles, grid emissions factors, and temperature. To determine the baseline energy consumption, it is necessary to separate the adjusted fuel economy values from temperature effects. This involves modifying the EPA [41] adjustments as outlined by Wu et al.[23]

For ICEVs, HEVs, and PHEVs in CS-mode, the following equations were used with fuel economy in miles/gallon for on-road adjustments excluding the effect of temperature:

$$FE_{g,city}^{No\,T} = \frac{1}{0.00187 + (\frac{1.134}{FE_{UDDS}})}$$

$$FE_{g,Highway}^{No\,T} = \frac{1}{0.00269 + (\frac{1.235}{FE_{HWFET}})}$$

For BEVs and PHEVs in CD-mode, the following equations were used with fuel economy in Wh/mile for on-road adjustments excluding the effect of temperature:

$$FE_{e,city}^{No\,T} = FE_{UDDS} + h_{city}(FE_{city}^{OR} - FE_{UDDS})$$

$$FE_{e,highway}^{No\,T} = FE_{HWFET} + h_{highway}(FE_{highway}^{OR} - FE_{HWFET})$$

Where h_{city}= 0.67 and $h_{highway}$ = 0.87.

Once temperature effects are separated from both city and highway fuel economy, we introduce a temperature-induced energy consumption adjustment factor. This adjustment factor is based on the last five full years of average monthly temperatures in each county in the U.S. (2019-2023) from NOAA.[45] To calculate the impact of temperature on fuel economy, we use the following piecewise function from Wu et al. [23], also used in Gan et al. [25]

$$r_{p,m} = \begin{cases} \alpha_{p,T_H} * (T_m - T_H) + 1, & \text{where } T_m > T_H \\ 1, & \text{where } T_c \leq T_m \leq T_H \\ \alpha_{p,T_C} * (T_C - T_m) + 1, & \text{where } T_m < T_C \end{cases}$$

Where α_{p,T_H} is the energy consumption adjustment for powertrain p at a temperature higher than the temperature T_H, and α_{p,T_c} is the energy consumption adjustment for powertrain p at a temperature lower than the temperature T_C. This adjustment factor is relative to the energy consumption for when the temperature is between T_C and T_H. This gives a monthly temperature adjusted fuel economy of $r_{p,m}$, which is then used to calculate a yearly adjustment.

$$r_{p,c} = \sum_{m=1}^{12} \frac{days\ in\ m}{365.25} * r_{p.m}$$

This is applied to each county. The constants used in these formulas are as follows:

$$T_C = 15.5\ ℃$$
$$\alpha_{ICEV,T_C} = 0.0064\ ℃^{-1}$$
$$\alpha_{HEV,T_C} = 0.0123\ ℃^{-1}$$
$$\alpha_{BEV,T_C} = 0.0242\ ℃^{-1}$$
$$T_H = 23.9\ ℃$$
$$\alpha_{ICEV,T_H} = 0.0129\ ℃^{-1}$$
$$\alpha_{HEV,T_H} = 0.0171\ ℃^{-1}$$
$$\alpha_{BEV,T_H} = 0.0210\ ℃^{-1}$$

We assume that when a PHEV is driving in charge sustaining mode it operates as an HEV (with the HEV temperature adjustment) and when a PHEV is driving in charge depleting mode it operates as a BEV (with the BEV temperature adjustment). The model from Wu et al. (2019) is based on empirical data including "on road testing data collected by Idaho National Laboratory's Advanced Vehicle project, on road Nissan Leaf data collected by FleetCarma, and on-road traveling data collected in the myFordMobile project by Ford Motor Company, and the dynamometer test database D3 from Argonne National Laboratory's Advanced Powertrain Research Facility."[23] This temperature model accounts for "significant cooling load" under hot conditions and "significant heating load and degraded battery performance or additional fuel use for cold starts" under cool conditions.[23]

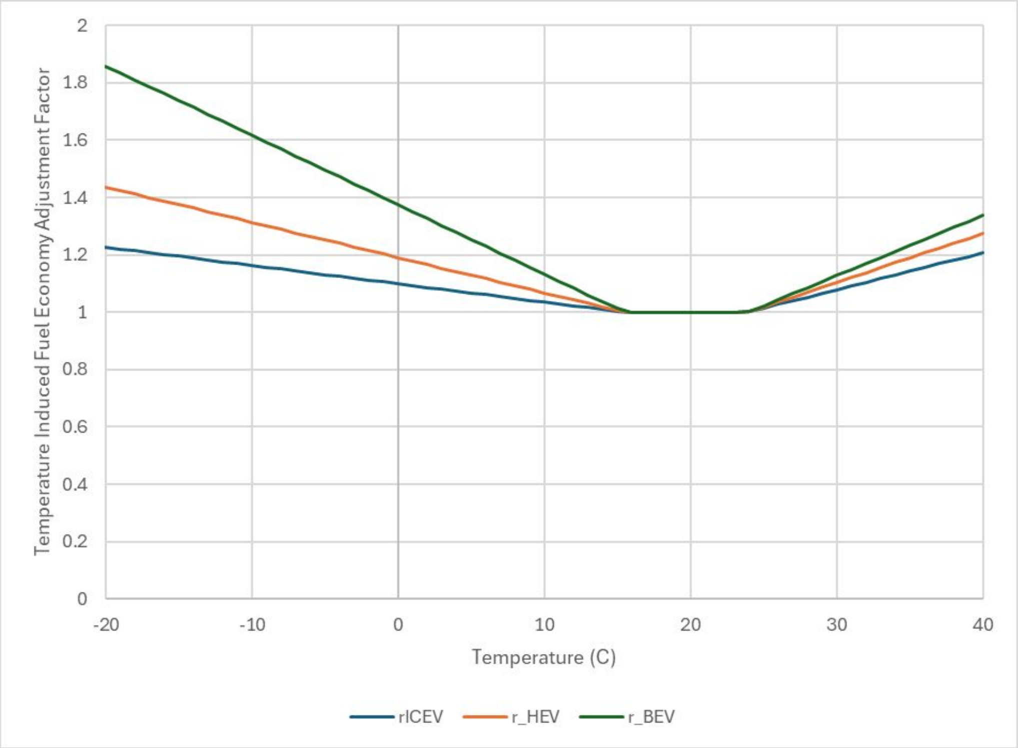

Figure S4. Temperature adjustment factor $r_{p,m}$

To calculate the GHG impact of BEVs and PHEVs operating in CD-mode we used the annual emissions factors from the NREL Cambium model.[39] The Cambium model breaks the contiguous US into 134 balancing areas and uses the outputs of two models to project future emissions factors: the Regional Energy Deployment (ReEDS) model, which projects structural changes to the US electricity grid using a least-cost framework under possible futures and the PLEXOS model which simulates commercial productions costs of future electric systems. The AER emissions factors range from 0 kg/MWh in Montana (BA 19) which has all its electricity needs met by hydroelectric and wind power generation [46] to 1000 kg/MWh in Arizona (BA 29) which has most of its energy demand met by coal. These emissions factors use a 100-year global warming potential for CH_4 and N_2O and include both combustion emissions and upstream emissions. The emissions factors are given in 5-year increments; we used linear interpolation to estimate emissions between years. Each county is assigned to a balancing area which can be seen in Figure S4. The emissions for the electric vehicles (BEV and PHEV-CD) are calculated using the following equation:

$$GHG_e = \sum_{y=2025}^{y+L_v} M_{v,y} * FE_{e,c} * EF_{c,y} / \eta$$

Most previous studies have used NERC regions (8 regions), eGRiD regions (22 regions) or states to account for the variability in emissions factors across the US.

Supplemental Note 6 – Fuel Reduction Values

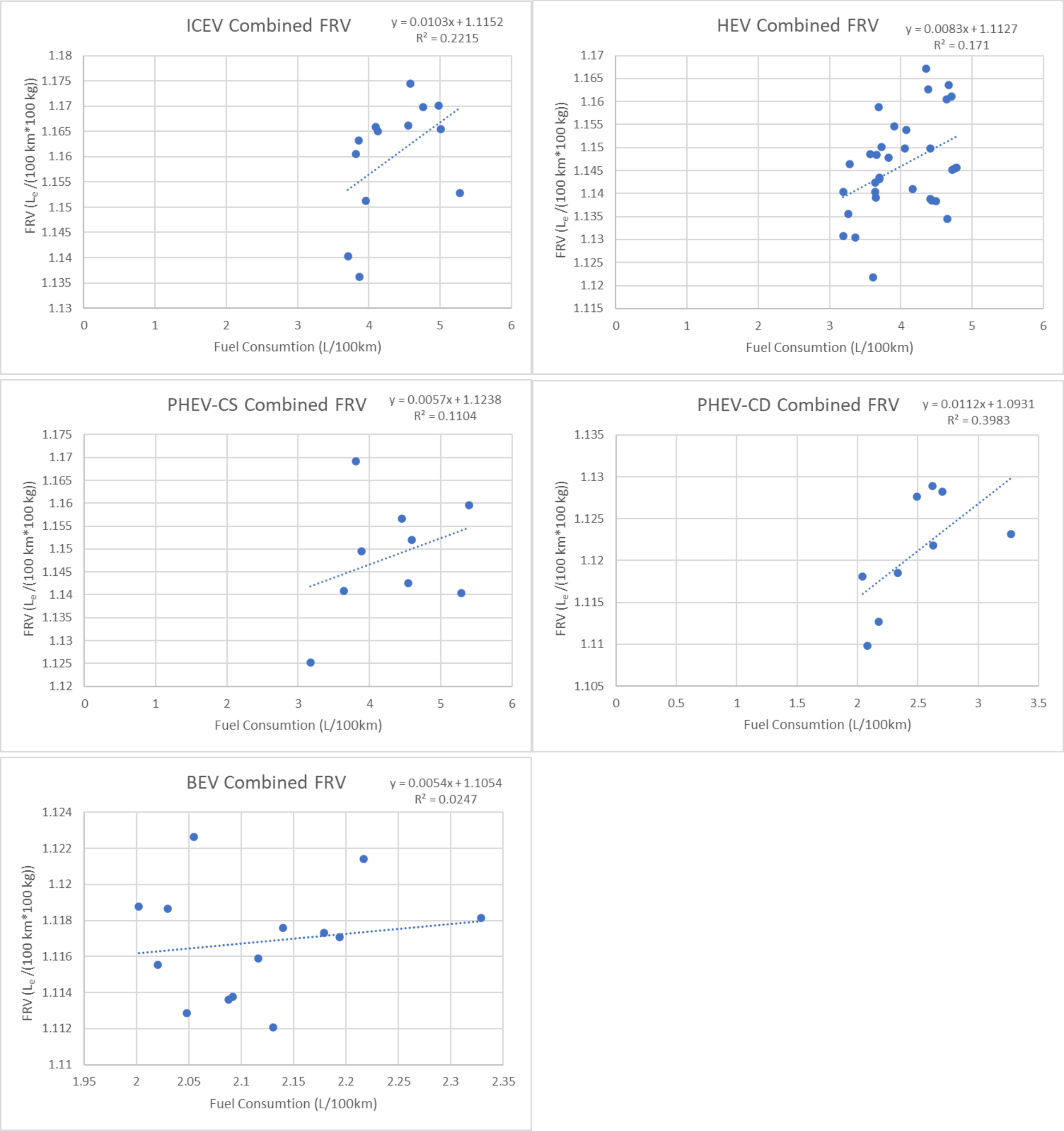

Figure S5. Linear regressions of data from Kim et al.

We utilized data from Kim et al. to derive the fuel reduction values (FRVs) incorporated into our model. [47] The key variables required for FRV calculation include vehicle mass, overall friction losses, and fuel consumption. We employed FRVs and fuel consumption data from 83 vehicle models in Chul et al. and applied linear regression to extrapolate the FRV for our generic vehicle model derived from Autonomie. The resulting linear regressions and FRVs are presented below.

Supplemental Note 7 – National Case Lifecycle Emissions

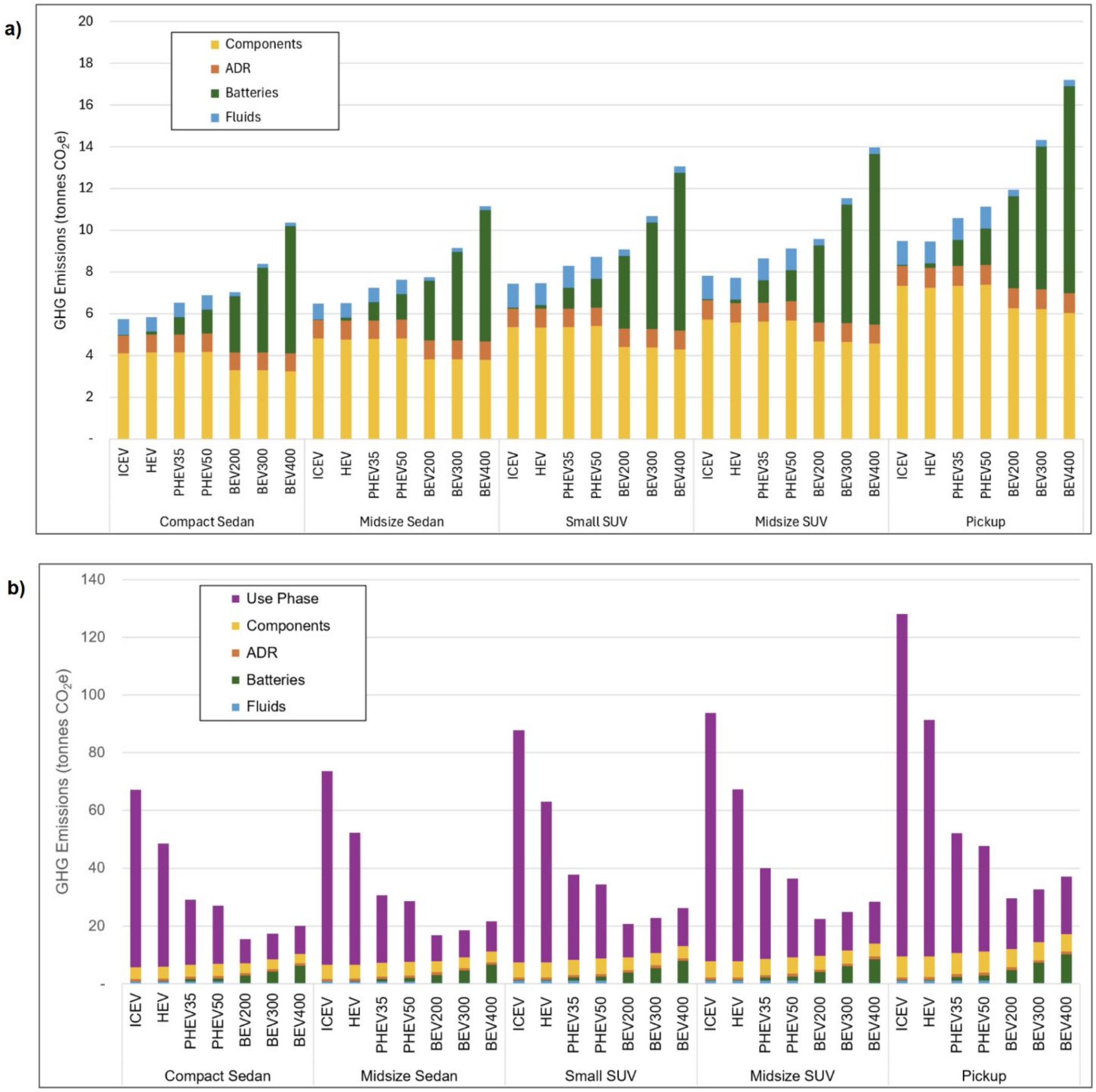

Figure S6. Greenhouse gas emissions for different powertrains and vehicle classes for a) vehicle cycle and b) total lifecycle. Vehicle cycle emissions include battery production, components, fluids, assembly, disposal, and recycling (ADR). For the PHEVs we assume the SAE standard utility factor.

To calculate lifecycle emissions, we start with the production burden and end of life, which are defined as the vehicle cycle emissions. These results are pulled from modified GREET output.[38] For the use phase calculations, we assume typical driving which is defined by a standard city/highway split of 43/57, SAE standard utility factors for PHEV defined by battery size (58% for PHEV35, 69% for PHEV50), a set VMT schedule, and no cargo.

The results for the compact sedan can be compared to those reported previously by Elgowainy et al.[18] The "current technology" and "future technology" results in Figure 2 from Elgowainy et al. show vehicle cycle emissions for a 2015 and a 2025-2030 model year compact sedan which can be compared to our results for a 2025 compact sedan shown in Figure S5a. As expected, given the general trend of increased use of renewable energy, the vehicle emissions we estimate for a 2025 compact sedan are generally lower (by about 10%) than those for a vehicle in 2015. The vehicle cycle emissions estimated for a 2025-2030 vehicle by Elgowainy et al. in 2018 are similar to those than we estimate for a 2025 vehicle.

We used NMC811 battery chemistry for HEVs, PHEVs, and BEVs. We examined the lifecycle emissions impact of different battery chemistries (NMC111, NMC532, NMC622, NMC955, and LFP) in Li-ion EVs using data entirely within the GREET model. The vehicle cycle, use-phase, and cradle-to-grave emissions for a 300-mile BEV SUV MY 2025 with different battery chemistries operated using 2030 U.S. grid average electricity are listed in Table S6. The total emissions for vehicles with NMC955, NMC622, NMC532, NMC111, and LFP batteries differ by less than 2.5% from those with NMC811.

Table S6. GREET Effects of Battery Chemistry on Lifecycle Emissions

	**Vehicle cycle**		**Well-to-pump (use phase)**		**Total**	
**Chemistry**	**Emissions (tonnes CO_2eq)**	**Relative to NMC811**	**Emissions (tonnes CO_2eq)**	**Relative to NMC811**	**Emissions (tonnes CO_2eq)**	**Relative to NMC811**
**NMC955**	10.79	-1.01%	16.06	-0.89%	28.85	-0.94%
**NMC811**	10.90	N/A	16.20	N/A	27.10	N/A
**NMC622**	10.95	+0.50%	16.46	1.59%	27.41	+1.15%
**NMC532**	10.88	-0.18%	16.49	1.78%	27.37	+0.99%
**NMC111**	11.04	+1.30%	16.74	3.30%	27.78	+2.49%
**LFP**	9.35	-14.26%	18.31	13.04%	27.66	+2.06%

Data from GREET 2024. Simulation year = 2030. Time lag = 5 years. Solid state synthesis for LFP. BEV 300 SUV Conventional EV.

Supplemental Note 8 – BEV Battery Size

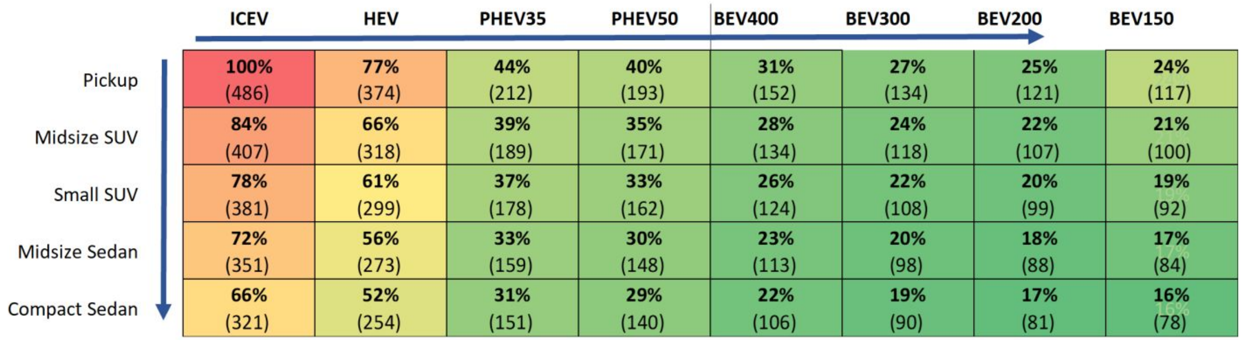

Figure S7. Expanded vehicle matrix with different BEV battery sizes.

Supplemental Note 9 – Electricity Emissions Factors

We also ran our model using the 100% decarbonization by 2035 scenario for a sensitivity analysis. The decarbonization scenario is the same as the mid-case except that emergent technologies are included and there is a constraint that forces a linear trajectory of the national electricity sector to net-zero by 2035. As can be seen in Figure S8, this scenario follows the mid-case very closely until 2030 and continues down to net zero by 2035. Because a higher proportion of driving is done in the earlier part of the lifetime of the vehicle, the differences between the mid-case and decarbonization scenario are not pronounced.

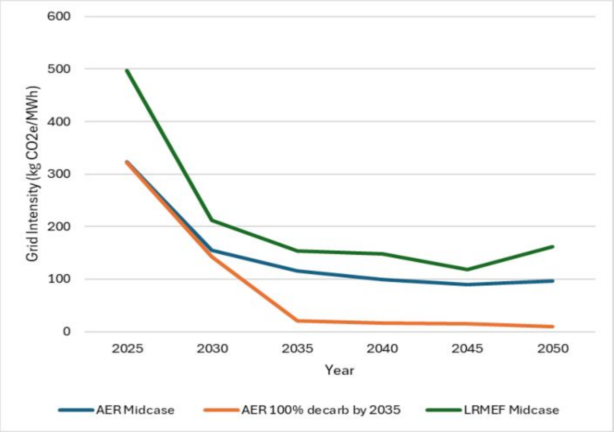

Figure S8. Annual electricity emissions factors for AER Midcase, AER 100% decarbonization by 2035 and LRMER Midcase

Supplemental Note 10 – Vehicle Miles Traveled

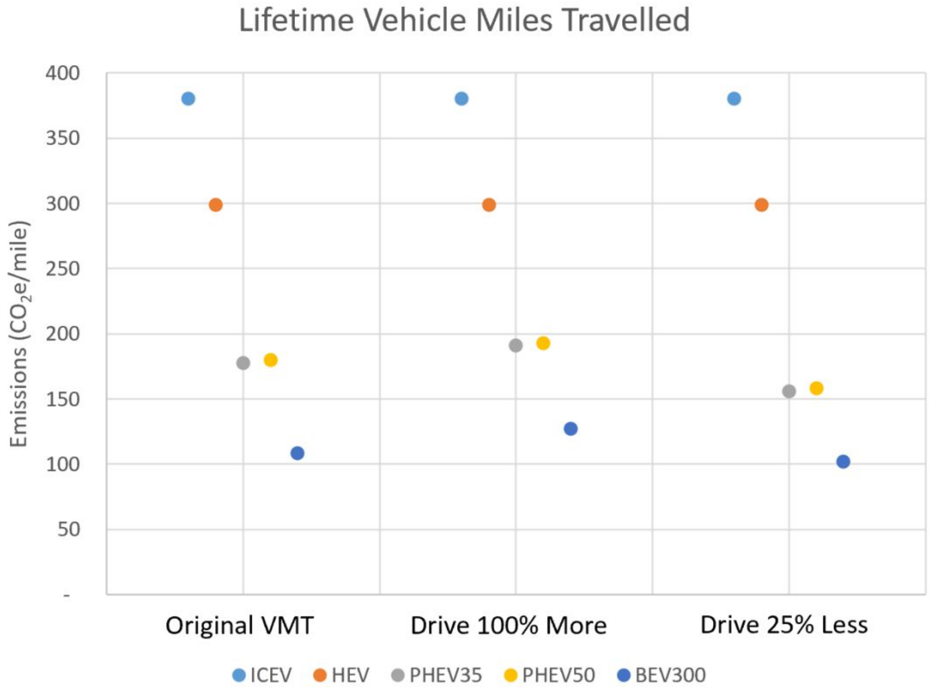

Figure S9. GHG emissions effected by different VMT driving patterns. Each scenario has the same total lifetime VMT, however the annual VMT is distributed based on the scenario over greater or fewer years.

Supplemental Note 11 – Vehicle Powertrain/Class Matrix

Delta in lifetime grams per mile switching from car in row to car in column

			To														
			ICEV					HEV					PHEV 35				
			Pickup	Midsize SUV	Small SUV	Midsize Sedan	Compact Sedan	Pickup	Midsize SUV	Small SUV	Midsize Sedan	Compact Sedan	Pickup	Midsize SUV	Small SUV	Midsize Sedan	Compact Sedan
		Pickup	0	-79	-105	-135	-165	-112	-168	-187	-213	-232	-272	-296	-307	-327	-335
		Midsize SUV	79	0	-26	-56	-86	-33	-89	-108	-134	-153	-193	-217	-228	-248	-256
	ICEV	Small SUV	105	26	0	-30	-60	-7	-63	-82	-108	-127	-167	-191	-202	-222	-230
		Midsize Sedan	135	56	30	0	-30	23	-33	-52	-78	-97	-137	-161	-172	-192	-200
		Compact Sedan	165	86	60	30	0	53	-3	-22	-48	-67	-107	-131	-142	-162	-170
		Pickup	112	33	7	-23	-53	0	-56	-75	-101	-120	-160	-184	-195	-215	-223
		Midsize SUV	168	89	63	33	3	56	0	-19	-45	-64	-104	-128	-139	-159	-167
	HEV	Small SUV	187	108	82	52	22	75	19	0	-26	-45	-85	-109	-120	-140	-148
		Midsize Sedan	213	134	108	78	48	101	45	26	0	-19	-59	-83	-94	-114	-122
		Compact Sedan	232	153	127	97	67	120	64	45	19	0	-40	-64	-75	-95	-103
		Pickup	272	193	167	137	107	160	104	85	59	40	0	-24	-35	-55	-63
		Midsize SUV	296	217	191	161	131	184	128	109	83	64	24	0	-11	-31	-39
	PHEV 35	Small SUV	307	228	202	172	142	195	139	120	94	75	35	11	0	-20	-28
		Midsize Sedan	327	248	222	192	162	215	159	140	114	95	55	31	20	0	-8
		Compact Sedan	335	256	230	200	170	223	167	148	122	103	63	39	28	8	0
		Pickup	291	212	186	156	126	179	123	104	78	59	19	-5	-16	-36	-44
		Midsize SUV	313	234	208	178	148	201	145	126	100	81	41	17	6	-14	-22
From	PHEV 50	Small SUV	323	244	218	188	158	211	155	136	110	91	51	27	16	-4	-12
		Midsize Sedan	337	258	232	202	172	225	169	150	124	105	65	41	30	10	2
		Compact Sedan	345	266	240	210	180	233	177	158	132	113	73	49	38	18	10
		Pickup	365	286	260	230	200	253	197	178	152	133	93	69	58	38	30
		Midsize SUV	380	301	275	245	215	268	212	193	167	148	108	84	73	53	45
	BEV 200	Small SUV	388	309	283	253	223	276	220	201	175	156	116	92	81	61	53
		Midsize Sedan	398	319	293	263	233	286	230	211	185	166	126	102	91	71	63
		Compact Sedan	405	326	300	270	240	293	237	218	192	173	133	109	98	78	70
		Pickup	352	273	247	217	187	240	184	165	139	120	80	56	45	25	17
		Midsize SUV	368	289	263	233	203	256	200	181	155	136	96	72	61	41	33
	BEV 300	Small SUV	378	299	273	243	213	266	210	191	165	146	106	82	71	51	43
		Midsize Sedan	389	310	284	254	224	277	221	202	176	157	117	93	82	62	54
		Compact Sedan	396	317	291	261	231	284	228	209	183	164	124	100	89	69	61
		Pickup	334	255	229	199	169	222	166	147	121	102	62	38	27	7	-1
		Midsize SUV	368	289	263	233	203	256	200	181	155	136	96	72	61	41	33
	BEV 400	Small SUV	378	299	273	243	213	266	210	191	165	146	106	82	71	51	43
		Midsize Sedan	389	310	284	254	224	277	221	202	176	157	117	93	82	62	54
		Compact Sedan	396	317	291	261	231	284	228	209	183	164	124	100	89	69	61

			PHEV 50					BEV 200					BEV 300					BEV 400				
			Pickup	Midsize SUV	Small SUV	Midsize Sedan	Compact Sedan	Pickup	Midsize SUV	Small SUV	Midsize Sedan	Compact Sedan	Pickup	Midsize SUV	Small SUV	Midsize Sedan	Compact Sedan	Pickup	Midsize SUV	Small SUV	Midsize Sedan	Compact Sedan
		Pickup	-291	-313	-323	-337	-345	-365	-380	-388	-398	-405	-352	-368	-378	-389	-396	-334	-352	-362	-374	-381
		Midsize SUV	-212	-234	-244	-258	-266	-286	-301	-309	-319	-326	-273	-289	-299	-310	-317	-255	-273	-283	-295	-302
	ICEV	Small SUV	-186	-208	-218	-232	-240	-260	-275	-283	-293	-300	-247	-263	-273	-284	-291	-229	-247	-257	-269	-276
		Midsize Sedan	-156	-178	-188	-202	-210	-230	-245	-253	-263	-270	-217	-233	-243	-254	-261	-199	-217	-227	-239	-246
		Compact Sedan	-126	-148	-158	-172	-180	-200	-215	-223	-233	-240	-187	-203	-213	-224	-231	-169	-187	-197	-209	-216
		Pickup	-179	-201	-211	-225	-233	-253	-268	-276	-286	-293	-240	-256	-266	-277	-284	-222	-240	-250	-262	-269
		Midsize SUV	-123	-145	-155	-169	-177	-197	-212	-220	-230	-237	-184	-200	-210	-221	-228	-166	-184	-194	-206	-213
	HEV	Small SUV	-104	-126	-136	-150	-158	-178	-193	-201	-211	-218	-165	-181	-191	-202	-209	-147	-165	-175	-187	-194
		Midsize Sedan	-78	-100	-110	-124	-132	-152	-167	-175	-185	-192	-139	-155	-165	-176	-183	-121	-139	-149	-161	-168
		Compact Sedan	-59	-81	-91	-105	-113	-133	-148	-156	-166	-173	-120	-136	-146	-157	-164	-102	-120	-130	-142	-149
		Pickup	-19	-41	-51	-65	-73	-93	-108	-116	-126	-133	-80	-96	-106	-117	-124	-62	-80	-90	-102	-109
		Midsize SUV	5	-17	-27	-41	-49	-69	-84	-92	-102	-109	-56	-72	-82	-93	-100	-38	-56	-66	-78	-85
	PHEV 35	Small SUV	16	-6	-16	-30	-38	-58	-73	-81	-91	-98	-45	-61	-71	-82	-89	-27	-45	-55	-67	-74
		Midsize Sedan	36	14	4	-10	-18	-38	-53	-61	-71	-78	-25	-41	-51	-62	-69	-7	-25	-35	-47	-54
		Compact Sedan	44	22	12	-2	-10	-30	-45	-53	-63	-70	-17	-33	-43	-54	-61	1	-17	-27	-39	-46
		Pickup	0	-22	-32	-46	-54	-74	-89	-97	-107	-114	-61	-77	-87	-98	-105	-43	-61	-71	-83	-90
		Midsize SUV	22	0	-10	-24	-32	-52	-67	-75	-85	-92	-39	-55	-65	-76	-83	-21	-39	-49	-61	-68
From	PHEV 50	Small SUV	32	10	0	-14	-22	-42	-57	-65	-75	-82	-29	-45	-55	-66	-73	-11	-29	-39	-51	-58
		Midsize Sedan	46	24	14	0	-8	-28	-43	-51	-61	-68	-15	-31	-41	-52	-59	3	-15	-25	-37	-44
		Compact Sedan	54	32	22	8	0	-20	-35	-43	-53	-60	-7	-23	-33	-44	-51	11	-7	-17	-29	-36
		Pickup	74	52	42	28	20	0	-15	-23	-33	-40	13	-3	-13	-24	-31	31	13	3	-9	-16
		Midsize SUV	89	67	57	43	35	15	0	-8	-18	-25	28	12	2	-9	-16	46	28	18	6	-1
	BEV 200	Small SUV	97	75	65	51	43	23	8	0	-10	-17	36	20	10	-1	-8	54	36	26	14	7
		Midsize Sedan	107	85	75	61	53	33	18	10	0	-7	46	30	20	9	2	64	46	36	24	17
		Compact Sedan	114	92	82	68	60	40	25	17	7	0	53	37	27	16	9	71	53	43	31	24
		Pickup	61	39	29	15	7	-13	-28	-36	-46	-53	0	-16	-26	-37	-44	18	0	-10	-22	-29
		Midsize SUV	77	55	45	31	23	3	-12	-20	-30	-37	16	0	-10	-21	-28	34	16	6	-6	-13
	BEV 300	Small SUV	87	65	55	41	33	13	-2	-10	-20	-27	26	10	0	-11	-18	44	26	16	4	-3
		Midsize Sedan	98	76	66	52	44	24	9	1	-9	-16	37	21	11	0	-7	55	37	27	15	8
		Compact Sedan	105	83	73	59	51	31	16	8	-2	-9	44	28	18	7	0	62	44	34	22	15
		Pickup	43	21	11	-3	-11	-31	-46	-54	-64	-71	-18	-34	-44	-55	-62	0	-18	-28	-40	-47
		Midsize SUV	77	55	45	31	23	3	-12	-20	-30	-37	16	0	-10	-21	-28	34	16	6	-6	-13
	BEV 400	Small SUV	87	65	55	41	33	13	-2	-10	-20	-27	26	10	0	-11	-18	44	26	16	4	-3
		Midsize Sedan	98	76	66	52	44	24	9	1	-9	-16	37	21	11	0	-7	55	37	27	15	8
		Compact Sedan	105	83	73	59	51	31	16	8	-2	-9	44	28	18	7	0	62	44	34	22	15

Figure S10. Vehicle powertrain/class matrix.

This matrix demonstrates relative GHG emissions of different powertrain and vehicle class combinations. This matrix is at a national level. We have built a database expanding this matrix into a database for each county in the continental US and under different use patterns (utility factor, drive cycle and cargo).

References

(1) Samaras, C.; Meisterling, K. Life Cycle Assessment of Greenhouse Gas Emissions from Plug-in Hybrid Vehicles: Implications for Policy. *Environ. Sci. Technol.* **2008**, *42* (9), 3170–3176. https://doi.org/10.1021/es702178s.

(2) Stephan, C. H.; Sullivan, J. Environmental and Energy Implications of Plug-In Hybrid-Electric Vehicles. *Environ. Sci. Technol.* **2008**, *42* (4), 1185–1190. https://doi.org/10.1021/es062314d.

(3) Jaramillo, P.; Samaras, C.; Wakeley, H.; Meisterling, K. Greenhouse Gas Implications of Using Coal for Transportation: Life Cycle Assessment of Coal-to-Liquids, Plug-in Hybrids, and Hydrogen Pathways. *Energy Policy* **2009**, *37* (7), 2689–2695. https://doi.org/10.1016/j.enpol.2009.03.001.

(4) Faria, R.; Marques, P.; Moura, P.; Freire, F.; Delgado, J.; De Almeida, A. T. Impact of the Electricity Mix and Use Profile in the Life-Cycle Assessment of Electric Vehicles. *Renew. Sustain. Energy Rev.* **2013**, *24*, 271–287. https://doi.org/10.1016/j.rser.2013.03.063.

(5) Graff Zivin, J. S.; Kotchen, M. J.; Mansur, E. T. Spatial and Temporal Heterogeneity of Marginal Emissions: Implications for Electric Cars and Other Electricity-Shifting Policies. *J. Econ. Behav. Organ.* **2014**, *107*, 248–268. https://doi.org/10.1016/j.jebo.2014.03.010.

(6) Lewis, A. M.; Kelly, J. C.; Keoleian, G. A. Vehicle Lightweighting vs. Electrification: Life Cycle Energy and GHG Emissions Results for Diverse Powertrain Vehicles. *Appl. Energy* **2014**, *126*, 13–20. https://doi.org/10.1016/j.apenergy.2014.03.023.

(7) Onat, N. C.; Kucukvar, M.; Tatari, O. Conventional, Hybrid, Plug-in Hybrid or Electric Vehicles? State-Based Comparative Carbon and Energy Footprint Analysis in the United States. *Appl. Energy* **2015**, *150*, 36–49. https://doi.org/10.1016/j.apenergy.2015.04.001.

(8) Tamayao, M.-A. M.; Michalek, J. J.; Hendrickson, C.; Azevedo, I. M. L. Regional Variability and Uncertainty of Electric Vehicle Life Cycle CO_2 Emissions across the United States. *Environ. Sci. Technol.* **2015**, *49* (14), 8844–8855. https://doi.org/10.1021/acs.est.5b00815.

(9) Orsi, F.; Muratori, M.; Rocco, M.; Colombo, E.; Rizzoni, G. A Multi-Dimensional Well-to-Wheels Analysis of Passenger Vehicles in Different Regions: Primary Energy Consumption, CO2

Emissions, and Economic Cost. *Appl. Energy* **2016**, *169*, 197–209. https://doi.org/10.1016/j.apenergy.2016.02.039.
(10) Holland, S. P.; Kotchen, M. J.; Mansur, E. T.; Yates, A. J. Why Marginal CO_2 Emissions Are Not Decreasing for US Electricity: Estimates and Implications for Climate Policy. *Proc. Natl. Acad. Sci.* **2022**, *119* (8), e2116632119. https://doi.org/10.1073/pnas.2116632119.
(11) Hoehne, C. G.; Chester, M. V. Optimizing Plug-in Electric Vehicle and Vehicle-to-Grid Charge Scheduling to Minimize Carbon Emissions. *Energy* **2016**, *115*, 646–657. https://doi.org/10.1016/j.energy.2016.09.057.
(12) McLaren, J.; Miller, J.; O'Shaughnessy, E.; Wood, E.; Shapiro, E. CO2 Emissions Associated with Electric Vehicle Charging: The Impact of Electricity Generation Mix, Charging Infrastructure Availability and Vehicle Type. *Electr. J.* **2016**, *29* (5), 72–88. https://doi.org/10.1016/j.tej.2016.06.005.
(13) Yuksel, T.; Tamayao, M.-A. M.; Hendrickson, C.; Azevedo, I. M. L.; Michalek, J. J. Effect of Regional Grid Mix, Driving Patterns and Climate on the Comparative Carbon Footprint of Gasoline and Plug-in Electric Vehicles in the United States. *Environ. Res. Lett.* **2016**, *11* (4), 044007. https://doi.org/10.1088/1748-9326/11/4/044007.
(14) Lombardi, L.; Tribioli, L.; Cozzolino, R.; Bella, G. Comparative Environmental Assessment of Conventional, Electric, Hybrid, and Fuel Cell Powertrains Based on LCA. *Int. J. Life Cycle Assess.* **2017**, *22* (12), 1989–2006. https://doi.org/10.1007/s11367-017-1294-y.
(15) Van Mierlo, J.; Messagie, M.; Rangaraju, S. Comparative Environmental Assessment of Alternative Fueled Vehicles Using a Life Cycle Assessment. *Transp. Res. Procedia* **2017**, *25*, 3435–3445. https://doi.org/10.1016/j.trpro.2017.05.244.
(16) Bicer, Y.; Dincer, I. Life Cycle Environmental Impact Assessments and Comparisons of Alternative Fuels for Clean Vehicles. *Resour. Conserv. Recycl.* **2018**, *132*, 141–157. https://doi.org/10.1016/j.resconrec.2018.01.036.
(17) De Souza, L. L. P.; Lora, E. E. S.; Palacio, J. C. E.; Rocha, M. H.; Renó, M. L. G.; Venturini, O. J. Comparative Environmental Life Cycle Assessment of Conventional Vehicles with Different Fuel Options, Plug-in Hybrid and Electric Vehicles for a Sustainable Transportation System in Brazil. *J. Clean. Prod.* **2018**, *203*, 444–468. https://doi.org/10.1016/j.jclepro.2018.08.236.
(18) Elgowainy, A.; Han, J.; Ward, J.; Joseck, F.; Gohlke, D.; Lindauer, A.; Ramsden, T.; Biddy, M.; Alexander, M.; Barnhart, S.; Sutherland, I.; Verduzco, L.; Wallington, T. J. Current and Future United States Light-Duty Vehicle Pathways: Cradle-to-Grave Lifecycle Greenhouse Gas Emissions and Economic Assessment. *Environ. Sci. Technol.* **2018**, *52* (4), 2392–2399. https://doi.org/10.1021/acs.est.7b06006.
(19) Karaaslan, E.; Zhao, Y.; Tatari, O. Comparative Life Cycle Assessment of Sport Utility Vehicles with Different Fuel Options. *Int. J. Life Cycle Assess.* **2018**, *23* (2), 333–347. https://doi.org/10.1007/s11367-017-1315-x.
(20) Kawamoto, R.; Mochizuki, H.; Moriguchi, Y.; Nakano, T.; Motohashi, M.; Sakai, Y.; Inaba, A. Estimation of CO2 Emissions of Internal Combustion Engine Vehicle and Battery Electric Vehicle Using LCA. *Sustainability* **2019**, *11* (9), 2690. https://doi.org/10.3390/su11092690.
(21) Desai, R. R.; Chen, R. B.; Hittinger, E.; Williams, E. Heterogeneity in Economic and Carbon Benefits of Electric Technology Vehicles in the US. *Environ. Sci. Technol.* **2019**, *54* (2), 1136–1146. https://doi.org/10.1021/acs.est.9b02874.
(22) Gai, Y.; Wang, A.; Pereira, L.; Hatzopoulou, M.; Posen, I. D. Marginal Greenhouse Gas Emissions of Ontario's Electricity System and the Implications of Electric Vehicle Charging. *Environ. Sci. Technol.* **2019**, *53* (13), 7903–7912. https://doi.org/10.1021/acs.est.9b01519.
(23) Wu, D.; Guo, F.; Field, F. R.; De Kleine, R. D.; Kim, H. C.; Wallington, T. J.; Kirchain, R. E. Regional Heterogeneity in the Emissions Benefits of Electrified and Lightweighted Light-Duty Vehicles. *Environ. Sci. Technol.* **2019**, *53* (18), 10560–10570. https://doi.org/10.1021/acs.est.9b00648.

(24) Tong, F.; Azevedo, I. M. L. What Are the Best Combinations of Fuel-Vehicle Technologies to Mitigate Climate Change and Air Pollution Effects across the United States? *Environ. Res. Lett.* **2020**, *15* (7), 074046. https://doi.org/10.1088/1748-9326/ab8a85.
(25) Gan, Y.; Lu, Z.; He, X.; Hao, C.; Wang, Y.; Cai, H.; Wang, M.; Elgowainy, A.; Przesmitzki, S.; Bouchard, J. Provincial Greenhouse Gas Emissions of Gasoline and Plug-in Electric Vehicles in China: Comparison from the Consumption-Based Electricity Perspective. *Environ. Sci. Technol.* **2021**, *55* (10), 6944–6956. https://doi.org/10.1021/acs.est.0c08217.
(26) Settey, T.; Gnap, J.; Synák, F.; Skrúcaný, T.; Dočkalik, M. Research into the Impacts of Driving Cycles and Load Weight on the Operation of a Light Commercial Electric Vehicle. *Sustainability* **2021**, *13* (24), 13872. https://doi.org/10.3390/su132413872.
(27) Woody, M.; Vaishnav, P.; Keoleian, G. A.; De Kleine, R.; Kim, H. C.; Anderson, J. E.; Wallington, T. J. The Role of Pickup Truck Electrification in the Decarbonization of Light-Duty Vehicles. *Environ. Res. Lett.* **2022**, *17* (3), 034031. https://doi.org/10.1088/1748-9326/ac5142.
(28) Reichmuth, D.; Dunn, J.; Anair, D. *Driving Cleaner: How Electric Cars and Pick-Ups Beat Gasoline on Lifetime Global Warming Emissions*; Report, Union of Concerned Scientists; 2022. https://www.ucsusa.org/resources/driving-cleaner.
(29) Kelly, J.; Elgowainy, A.; Isaac, R.; Ward, J.; Islam, E.; Rousseau, A.; Sutherland, I.; Wallington, T.; Alexander, M.; Muratori, M.; Franklin, M.; Adams, J.; Rustagi, N. *Cradle-to-Grave Lifecycle Analysis of U.S. Light-Duty Vehicle-Fuel Pathways: A Greenhouse Gas Emissions and Economic Assessment of Current (2020) and Future (2030-2035) Technologies*; ANL-22/27, 1875764, 176270; 2022; p ANL-22/27, 1875764, 176270. https://doi.org/10.2172/1875764.
(30) Rashid, S.; Pagone, E. Cradle-to-Grave Lifecycle Environmental Assessment of Hybrid Electric Vehicles. *Sustainability* **2023**, *15* (14), 11027. https://doi.org/10.3390/su151411027.
(31) Jenn, A. Emissions of Electric Vehicles in California's Transition to Carbon Neutrality. *Appl. Energy* **2023**, *339*, 120974. https://doi.org/10.1016/j.apenergy.2023.120974.
(32) Bruchon, M.; Chen, Z. L.; Michalek, J. Cleaning up While Changing Gears: The Role of Battery Design, Fossil Fuel Power Plants, and Vehicle Policy for Reducing Emissions in the Transition to Electric Vehicles. *Environ. Sci. Technol.* **2024**, *58* (8), 3787–3799. https://doi.org/10.1021/acs.est.3c07098.
(33) Singh, M.; Yuksel, T.; Michalek, J. J.; Azevedo, I. M. L. Ensuring Greenhouse Gas Reductions from Electric Vehicles Compared to Hybrid Gasoline Vehicles Requires a Cleaner U.S. Electricity Grid. *Sci. Rep.* **2024**, *14* (1), 1639. https://doi.org/10.1038/s41598-024-51697-1.
(34) Maselli, M.; Pelegrina, J.; Marotti De Mello, A.; Ribeiro Souza, J. V.; Marx, R.; Priarone, P. C. Electric or Internal Combustion Vehicles? A Life Cycle Assessment in São Paulo. *Renew. Sustain. Energy Rev.* **2025**, *212*, 115431. https://doi.org/10.1016/j.rser.2025.115431.
(35) Gagnon, P. J.; Bistline, J. E. T.; Alexander, M. H.; Cole, W. J. Short-Run Marginal Emission Rates Omit Important Impacts of Electric-Sector Interventions. *Proc. Natl. Acad. Sci.* **2022**, *119* (49), e2211624119. https://doi.org/10.1073/pnas.2211624119.
(36) Hawkes, A. D. Long-Run Marginal CO2 Emissions Factors in National Electricity Systems. *Appl. Energy* **2014**, *125*, 197–205. https://doi.org/10.1016/j.apenergy.2014.03.060.
(37) U.S. Environmental Protection Agency. Emissions & Generation Resource Integrated Database (eGRID), 2025. https://www.epa.gov/egrid (accessed 2025-03-03).
(38) Wang, M.; Elgowainy, A.; Lee, U.; Baek, K.; Balchandani, S.; Benavides, P.; Burnham, A.; Cai, H.; Chen, P.; Gan, Y.; Gracida-Alvarez, U.; Hawkins, T.; Huang, T.-Y.; Iyer, R.; Kar, S.; Kelly, J.; Kim, T.; Kolodziej, C.; Lee, K.; Liu, X.; Lu, Z.; Masum, F.; Morales, M.; Ng, C.; Ou, L.; Poddar, T.; Reddi, K.; Shukla, S.; Singh, U.; Sun, L.; Sun, P.; Sykora, T.; Vyawahare, P.; Zhang, J. Greenhouse Gases, Regulated Emissions, and Energy Use in Technologies Model ® (2023 Excel), 2023. https://doi.org/10.11578/GREET-EXCEL-2023/DC.20230907.1.
(39) Gagnon, P.; Perez, P. A. S.; Obika, K.; Schwarz, M.; Morris, J.; Gu, J.; Eisenman, J. Cambium 2023 Scenario Descriptions and Documentation. *Renew. Energy* **2024**.

(40) Ehsan Sabri Islam; Daniela Nieto Prada; Ram Vijayagopal; Charbel Mansour; Paul Phillips; Namdoo Kim; Michel Alhajjar. *Detailed Simulation Study to Evaluate Future Transportation Decarbonization Potential*; Argonne National Lab, 2023. https://publications.anl.gov/anlpubs/2023/11/186057.pdf (accessed 2024-08-21).
(41) EPA. *Fuel Economy*. https://www.fueleconomy.gov/feg/PowerSearch.do?action=PowerSearch&year1=2023&year2=2024&minmsrpsel=0&maxmsrpsel=0&city=0&highway=0&combined=0&YearSel=2023-2024&MakeSel=&MarClassSel=&FuelTypeSel=&VehTypeSel=&TranySel=&DriveTypeSel=&CylindersSel=&MpgSel=&sortBy=&Units=&url=SearchServlet&opt=new&minmsrp=0&maxmsrp=0&minmpg=&maxmpg=&sCharge=&tCharge=&startstop=&cylDeact=&rowLimit=50 (accessed 2024-09-25).
(42) Hamza, K.; Laberteaux, K. P. Utility Factor Curves for Plug-in Hybrid Electric Vehicles: Beyond the Standard Assumptions. *World Electr. Veh. J.* **2023**, *14* (11), 301. https://doi.org/10.3390/wevj14110301.
(43) NHTSA. *Final Rulemaking for Model Years 2024-2026 LightDuty Vehicle Corporate Average Fuel Economy Standards*; 2022. https://www.nhtsa.gov/sites/nhtsa.gov/files/2022-04/Final-TSD_CAFE-MY-2024-2026.pdf (accessed 2024-08-20).
(44) Amgad Elgowainy; Jeongwoo Han; Jacob Ward; Fred Joseck; David Gohike; Alicia Lindauer; Todd Ramsden; Mary Biddy; Marcus Alexander; Steven Barnhart; Ian Sutherland; Larua Verduzco; Timothy Wallington. *Cradle-to-Grave Lifecycle Analysis of U.S. Light-Duty Vehicle-Fuel Pathways: A Greenhouse Gas Emissions and Economic Assessment of Current (2015) and Future (2025-2030) Technologies.*; 2016. https://publications.anl.gov/anlpubs/2016/09/130244.pdf (accessed 2024-09-25).
(45) NOAA. Climate at a Glance | County Mapping | National Centers for Environmental Information (NCEI), 2023. https://www.ncei.noaa.gov/access/monitoring/climate-at-a-glance/county/mapping/110/tavg/202306/60/value (accessed 2024-08-21).
(46) *U.S. Energy Information Administration - EIA - Independent Statistics and Analysis*. https://www.eia.gov/state/analysis.php?sid=MT (accessed 2024-09-25).
(47) Kim, H. C.; Wallington, T. J.; Sullivan, J. L.; Keoleian, G. A. Life Cycle Assessment of Vehicle Lightweighting: Novel Mathematical Methods to Estimate Use-Phase Fuel Consumption. *Environ. Sci. Technol.* **2015**, *49* (16), 10209–10216. https://doi.org/10.1021/acs.est.5b01655.
